# Electrochemical Evaluation of Caffeic Acid as an Eco-Friendly Corrosion Inhibitor for Brass and Copper in NaCl

**DOI:** 10.3390/molecules31213837

**Published:** 2026-10-23

**Authors:** Aleksander Kucharek, Elżbieta Kuśmierek, Aleksandra Bednarek, Aleksandra Zimon, Waldemar Maniukiewicz, Kashif Mairaj Deen, Małgorzata Iwona Szynkowska-Jóźwik

**Affiliations:** 1 Institute of General and Ecological Chemistry, Lodz University of Technology, ul. Zeromskiego 116, 90-924 Lodz, Polandaleksandra.zimon@p.lodz.pl (A.Z.); waldemar.maniukiewicz@p.lodz.pl (W.M.);; 2 Department of Materials Engineering, The University of British Columbia, Vancouver, BC V6T 1Z4, Canada; kashifmairaj.deen@ubc.ca

**Keywords:** green corrosion inhibitor, caffeic acid, copper corrosion, brass corrosion, electrochemical studies, surface morphology

## Abstract

Caffeic acid (CA) is one of the green corrosion inhibitors that can be efficiently applied in protection of metallic materials. Its inhibition properties were investigated for Cu and brass in 0.1 M NaCl solution using electrochemical techniques. The anticorrosion properties of CA was tested by applying coatings from its ethanol solutions and, for comparison, by adding CA directly to the corrosive medium. The effects of varying number of coating layers deposited from CA solutions of different concentrations were examined. The development of a durable coating that does not affect the appearance of the protected material could enable this method to be used for corrosion protection of historical heritage artefacts made of copper and brass. Potentiodynamic polarization (PDP) and electrochemical impedance spectroscopy (EIS) measurements proved that CA applied in the form of coatings on Cu and brass was a more efficient protection method than modifying corrosive medium with this inhibitor. Increasing immersion time resulted in a better corrosion protection in the case of CA coating. The highest inhibition efficiency was achieved with 20 layers formed from 5 mM CA solution (86.5%) and 20 layers formed from 10 mM CA solution (81.4%) for Cu and brass, respectively. EIS measurements revealed an influence of diffusion on the corrosion rate both in the modified medium and in the case of CA coatings. The effectiveness of CA coatings on Cu and brass corrosion was further confirmed by SEM-EDS, XRD and TOF-SIMS measurements as well as experiments performed in a salt spray chamber.

## 1. Introduction

Copper is without a doubt one of the most crucial metals in many different branches of industry, such as electronics, pipeline systems, military, transportation, or construction [1,2,3]. Its wide range of applications can be attributed to exceptional thermal and electrical conductivity, machinability, workability, and good corrosion resistance [4]. However, under certain conditions, e.g., the presence of Cl^−^ or SO_4_^−^ ions, Cu can undergo pitting corrosion, which can lead to serious damages [5]. Apart from the aforementioned applications, it is equally important that Cu is a component of brass. Similarly to Cu, brass is generally resistant to corrosion; however, prolonged exposure to atmospheric corrosion can result in the occurrence of a type of selective corrosion called dezincification, which can cause the drastic destruction of this alloy [6,7]. Brass is a material that a significant number of historic heritage artefacts are made of [8,9]. In the case of such objects, corrosion is considerably a more severe issue, due to the cultural value being lost without the ability to recover it. Taking all of the above into consideration, it is obvious that corrosion protection of these materials is an extremely important task.

One of the most common methods of protecting metals and alloys against corrosion is applying corrosion inhibitors [10,11,12]. The danger connected to this method is the fact that classic corrosion inhibitors are toxic both to humans and to the environment [13,14]. In order to address this issue, an increasing number of safe and ecological corrosion inhibitors (so-called green corrosion inhibitors) have been introduced recently. Caffeic acid, which is a derivative of cinnamic acid, is a great example of such an inhibitor. It is known that this compound quite commonly occurs in nature. It can be found in a variety of plants, e.g., rice, coffee, apples, or plums [15,16,17]. It is also worth noting that caffeic acid, in many different ways, positively impacts human health. For example, it exhibits anti-inflammatory, cardioprotective or neuroprotective properties [18]. Green corrosion inhibitors have already presented excellent anticorrosive properties, on par with classic inhibitors and have been applied for copper [19,20,21,22,23], brass [19,24,25,26,27], various steels [28,29,30,31,32] or aluminum and its alloys [33,34,35,36,37]. Carbon quantum dots are an interesting example of green corrosion inhibitors applied for protection of copper–nickel alloys in H_2_SO_4_ solution [38,39] and magnesium alloy in NaCl solution [40,41]. They were obtained using biomass feedstocks, soybean residue, sesame straw or biodegradable plastic bags. Moreover, extracts from naturally occurring plants were investigated as green corrosion inhibitors for Cu in acidic media (H_2_SO_4_) [42,43,44,45]. Brass anticorrosion protection with the application of leaf or fruit extracts was also investigated in harsh media, e.g., HCl, HNO_3_, H_2_SO_4_ or seawater [46,47,48,49]. The performed literature review demonstrates that caffeic acid has not been widely described as a corrosion inhibitor. Thus far, there have been reports on applying this corrosion inhibitor in protection of Zn [50], Cu (only as a corrosive medium modification) [51], mild steel [52] and Mg alloy protection [53].

In this work, the impact of caffeic acid on the corrosion of Cu and brass in NaCl was studied. It was confirmed that Cl^−^ ions accelerate the corrosion of both Cu and brass. Coatings deposited from caffeic acid solutions seem to be a promising method of protecting historic heritage artefacts since they do not have any effect on the color or the appearance of the protected materials. CA as a corrosion inhibitor for Cu and brass was investigated both by modifying the corrosive medium and by applying CA protective coatings on the tested samples’ surfaces. Investigations were conducted using electroanalytical methods such as potentiodynamic polarization (PDP) with OCP (open circuit potential) measurement and EIS (electrochemical impedance spectroscopy). Furthermore, SEM-EDS (scanning electron microscopy coupled with energy dispersive spectroscopy), XRD (X-ray diffraction) and TOF-SIMS (time-of-flight secondary ion mass spectrometry) methods were used in the analysis of changes in morphology of Cu and brass samples before and after immersion in the corrosive medium both with and without protective coatings. The results obtained in this study revealed that applying CA as a green corrosion inhibitor for Cu and brass has great potential.

## 2. Results

### 2.1. Electrochemical Studies

PDP curves were recorded for pure Cu and brass in 0.1 M NaCl in three consecutive experiments with a 1 h OCP measurement taken prior to the experiments. Measurements in 0.1 M NaCl modified by the addition of CA at different concentrations and for tested samples with applied CA coatings were conducted in a similar manner. The linear Tafel fitting was performed on the PDP curves, which enabled determining the corrosion parameters. A cathodic part of Tafel plots is attributed to the reduction of dissolved oxygen according to Reaction (1) [54,55]:

O_2_ + 2H_2_O + 4e^−^ → 4OH^−^ (1)

An anodic part of Tafel plots on the other hand is connected to the copper dissolution according to the following reactions [54]:

Cu → Cu^+^ + e^−^ (2)

Cu^+^ + Cl^−^ → CuCl (3)

CuCl + Cl^−^ → CuCl_2_^−^ (4)

CuCl_2_^−^ → Cu^2+^ + 2Cl^−^ + e^−^ (5)

or CuCl_2_^−^ can undergo a following hydrolysis [56,57]:

2CuCl_2_^−^ + 2OH^−^ → Cu_2_O + 4Cl^−^ + H_2_O (6)

In the case of brass, immersing this material in NaCl solution results in its corrosion proceeding in two steps. In the first step the formation of zinc oxide layers occurs via the following reactions [55]:

Zn + O_2_ + 4H^+^ + 2e^−^→ Zn^2+^ + 2H_2_O (7)

Zn^2+^ + H_2_O → ZnO + 2H^+^ (8)

Reactions (7) and (8) result in dezincification process and are followed by enrichment of the brass surface with copper. In the second step, chlorides penetrate through zinc corrosion products and enhance the corrosion of copper, resulting in the formation of Cu_2_O, according to Reactions (9)–(11) [58]:

Cu → Cu^+^ + e^−^ (9)

Cu^+^ → Cu^2+^ + e^−^ (10)

2Cu^+^ + H_2_O → Cu_2_O + 2H^+^ (11)

Subsequently, due to the presence of Cl^−^, the formation of CuCl can proceed according to Reaction (3). Due to the poor adhesive properties of this layer, its protective abilities are insufficient. Longer immersion in NaCl causes this layer to be transformed into CuCl_2_^−^, via Reaction (4).

#### 2.1.1. Corrosion Inhibition via CA Addition

**Figure 1:**
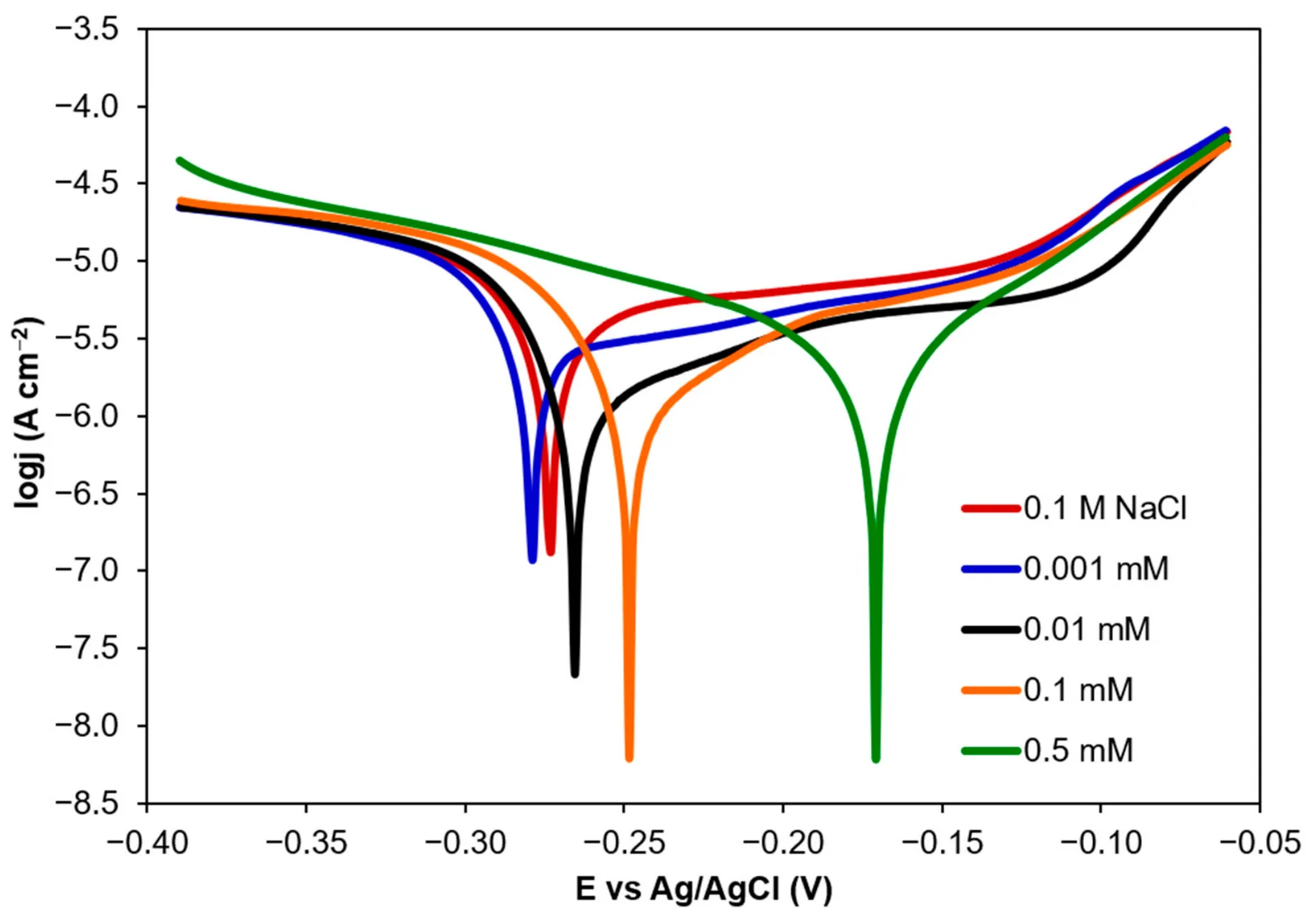
Exemplary PDP curves recorded for Cu in 0.1 M NaCl modified by the addition of CA at different concentrations.

**Figure 2:**
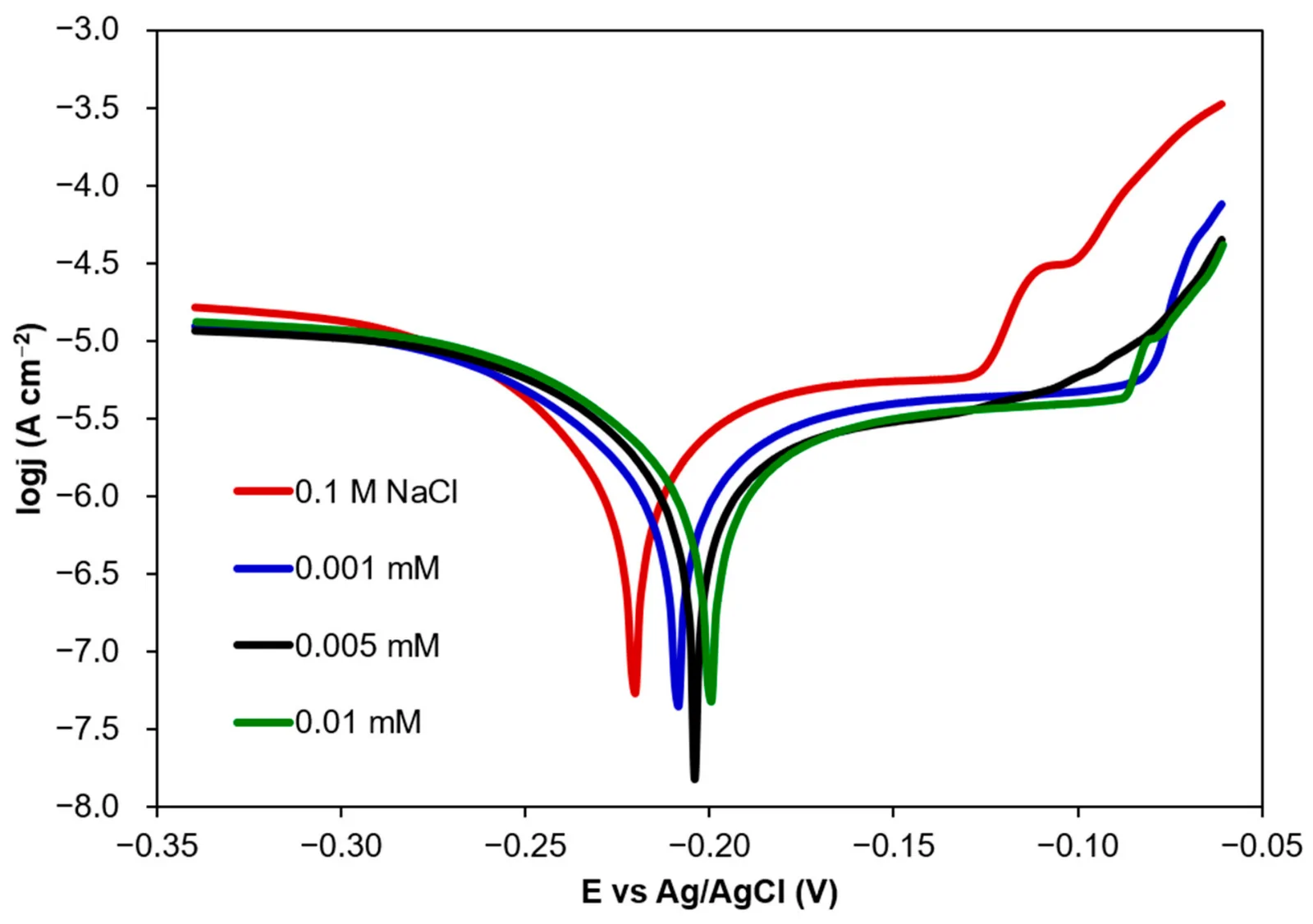
Exemplary Tafel curves recorded for brass in 0.1 M NaCl modified by the addition of CA at various concentrations.

The inhibitory properties of CA for Cu and brass in the presence of Cl^−^ ions were tested by recording PDP curves in NaCl solution modified by CA at different concentrations. Exemplary Tafel plots recorded for Cu and brass are presented in Figure 1 and Figure 2, respectively.

The corrosion parameters obtained from PDP measurements were used to calculate the corrosion rate (CR) and inhibition efficiency (IE). Their values are shown in Table 1 and Table 2.

Figure 1 shows that the current density (j) in the cathodic and anodic branch of PDP curves is decreased in the presence of CA. This proves that CA acts as the corrosion inhibitor and decreases the rate of both anodic and cathodic reactions. The changes in the shape of cathodic and anodic branches are slight but changes in the current density are higher for anodic branches. This proves that CA suppresses mainly the anodic reaction. Additionally, all anodic branches present the plateau region in the potential range ca. −0.20 to −0.10 V except for curve recorded for CA concentration of 0.5 mM. This plateau may be attributed to CA adsorption on the sample surface and formation of a protective film [59,60] resulting in passivation phenomena and suppression of anodic dissolution of Cu. Further increase in the polarization potential results in the increase in the current density due to deterioration in the stability of the protective adsorption film and facilitating anodic dissolution of Cu [61]. In the case of a PDP curve recorded for NaCl solution, a plateau region is also observed. This plateau may be attributed to formation of a film containing corrosion products. This film may also protect against corrosion. However, the current density in this plateau region is higher in comparison with solutions containing CA and indicates worse protection against Cu corrosion.

Similarly, as it was observed that in the case of Cu, PDP curves recorded for brass samples (Figure 2) show a higher decrease in the current density of anodic branches in comparison to cathodic branches recorded in the presence of CA. This proves a slightly higher effect of CA on anodic dissolution of brass components. All anodic branches are characterized by the plateau region in the potential range from ca. −0.18 to −0.08 V. This plateau may also be attributed to formation of CA film adsorbed on the sample surface causing passivation phenomena. At higher potentials, there is a visible increase in the current density resulting from deterioration of CA film stability and facilitation of the anodic dissolution of brass components. The plateau region observed in NaCl solution without CA addition may also be attributed to formation of corrosion product film which can only partially protect against corrosion. The current density in this plateau region is clearly higher in comparison with solutions containing CA. Taking into consideration its value as well as lower potential (ca. −0.125 V) at which its increase is observed, it may be assumed that the film containing corrosion products reveals worse protection against brass corrosion.

In the case of both tested materials, there was an optimal concentration of added CA deduced from PDP measurements (Table 1 and Table 2). For Cu, the highest value of IE (71.5%) was observed for CA concentration of 0.1 mM, similarly as it was achieved for Zn samples (82%) described in our previous paper [50]. However, slightly higher optimal value of IE (72.5%) was obtained for brass for CA concentration 10 times lower (0.01 mM).

A comparison of E_corr_ values determined for Cu and brass in the presence and absence of CA indicates the adsorption of the inhibitor on the tested samples surfaces can be attributed to the so-called “geometric blocking effect”, but alteration of activation energy cannot be excluded [51]. Moreover, E_corr_ values were shifted towards more positive values by no more than 25 mV for Cu (apart from the highest CA concentration) and by 20 mV for brass. Because changes in E_corr_ values were not higher than 85 mV, it can be concluded that CA acts as a mixed-type corrosion inhibitor, retarding both anodic and cathodic reactions [62,63]. The analysis of anodic Tafel slope values (b_a_) revealed a significant decrease for Cu in the presence of CA. This suggests that the CA inhibition mechanism is ascribed mainly to suppressing the dissolution of the metal [64,65]. Furthermore, the corrosion mechanism is probably changed by formation of basic copper chloride [66] as an intermediate corrosion product. For brass, on the other hand, the values of b_a_ show very little differences, indicating that the corrosion anodic mechanism remains unchanged [67,68]. Cathodic Tafel slope values (b_c_) only slightly changed for both tested materials, regardless of the CA concentration. Nevertheless, the lowest values of b_a_ and b_c_ were observed for the optimal concentrations of CA. The decrease in IE for higher CA concentrations is accompanied by the increase in Tafel slope values. This can be ascribed to the oversaturation of CA molecules, which may result in either desorption or poor adsorption of the inhibitor [69].

The decrease in j_corr_ values can be attributed to the adsorption of CA on Cu and brass surfaces. The coverage of metal surfaces by corrosion inhibitors is a parameter closely related to the inhibition efficiency. The degree of surface coverage (Θ) by inhibitors is a parameter closely related to the inhibition efficiency and can be calculated from the following equation [70]:

(12) $$\theta =\frac{\mathrm{IE}}{100}$$

Parameters obtained from PDP measurements were used to fit the appropriate adsorption isotherm. It was concluded that the best fitting for the analyzed data was obtained for the Langmuir isotherm both for Cu and brass. Data for the second component of brass–Zn were also fitted by the Langmuir isotherm [50], which is given by the following equation [71]:

(13) $$\frac{C}{\theta }=\frac{1}{K_{\mathrm{ads}}}+C$$

**Figure 3:**
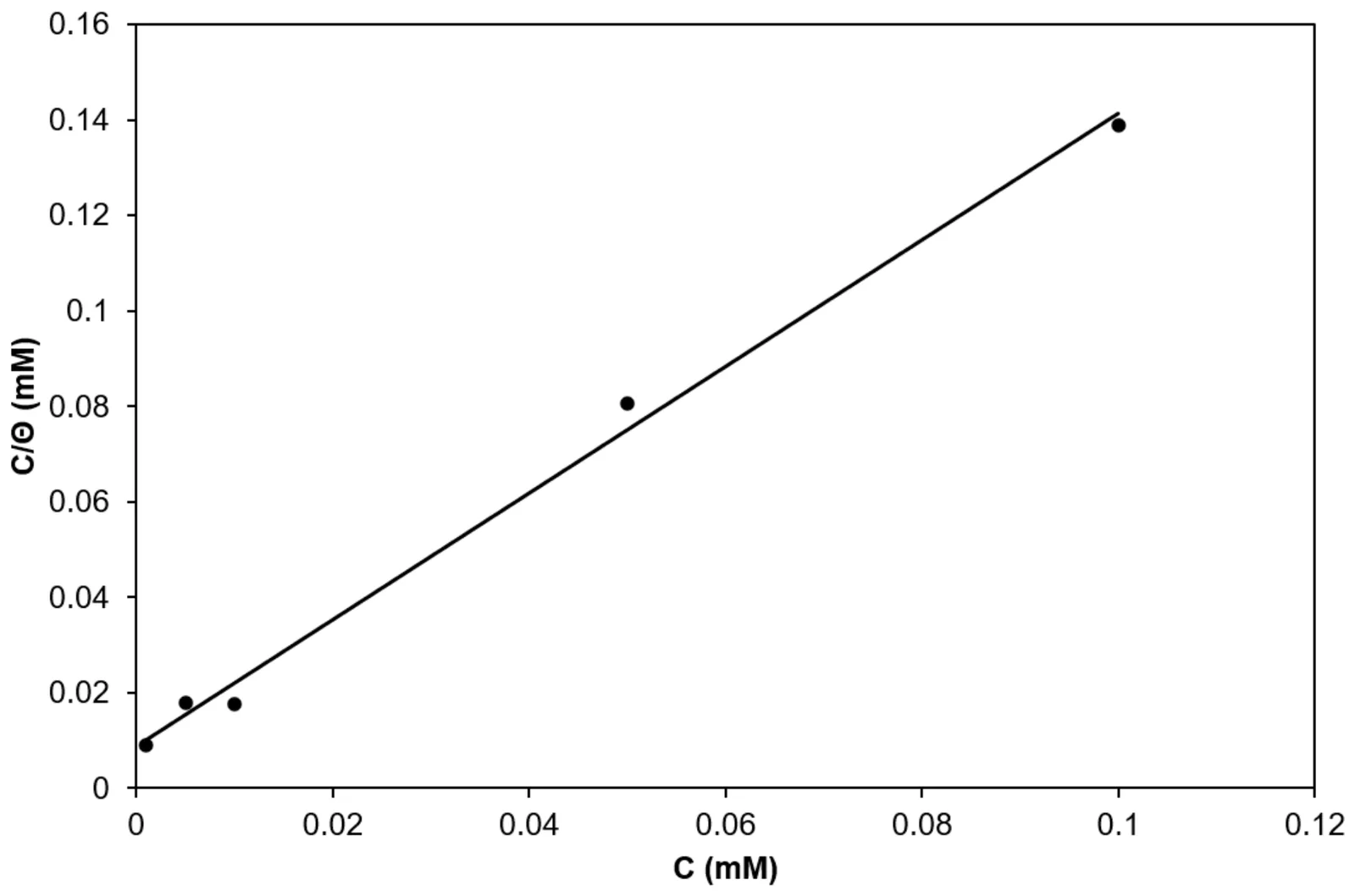
Langmuir isotherm for CA on Cu surface in 0.1 M NaCl.

**Figure 4:**
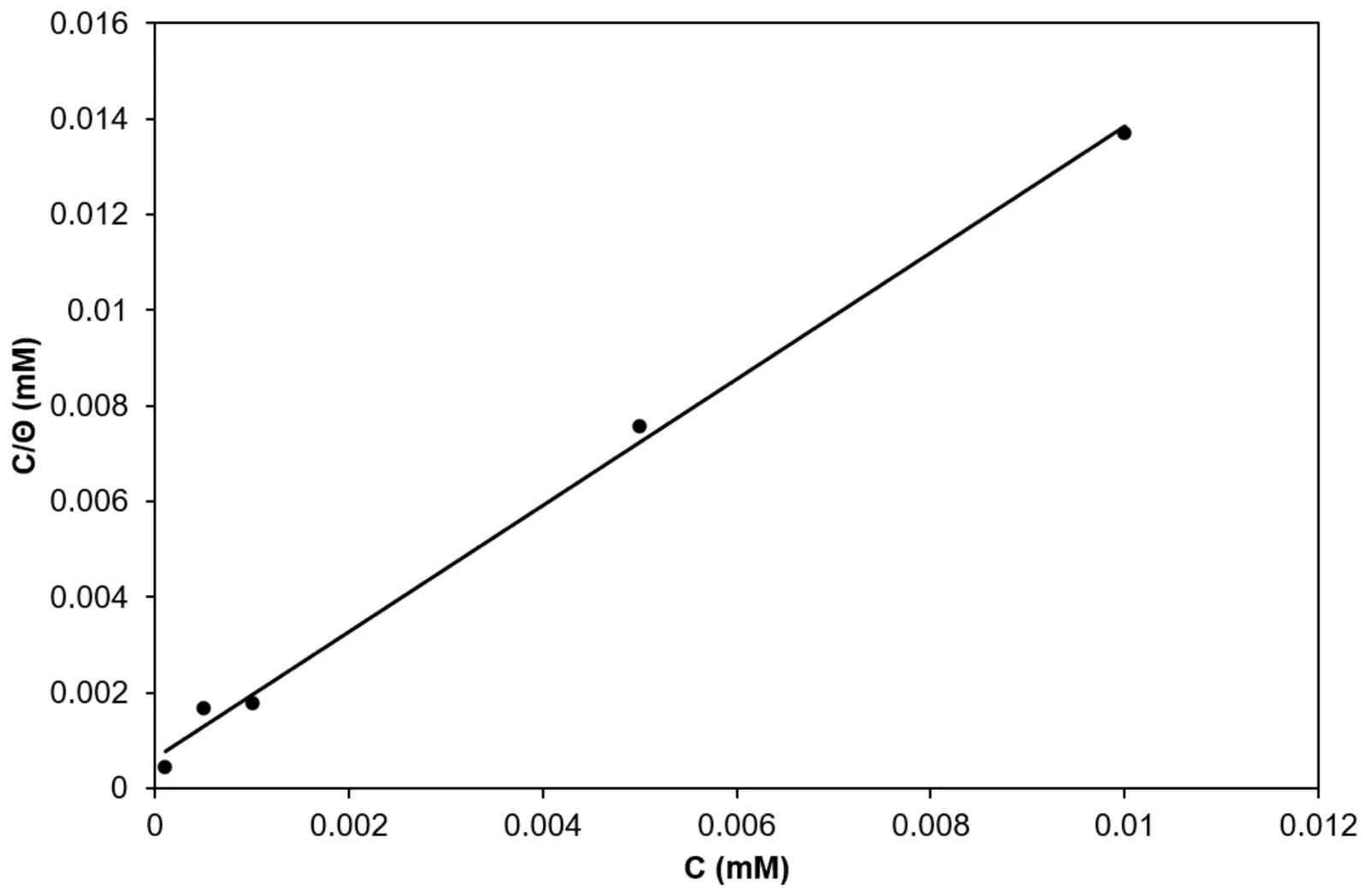
Langmuir isotherm for CA on brass surface in 0.1 M NaCl.

The C/Θ vs. C (C-CA concentration) plots are shown in Figure 3 and Figure 4 and are both straight lines with a high correlation coefficient (R) value close to unity.

However, the Langmuir isotherm can be fitted only to the optimal concentration of CA for both materials, i.e., 0.1 mM and 0.01 mM for Cu and brass, respectively. At higher concentrations of CA, probably due to the oversaturation of tested samples surfaces with the inhibitor molecules, intermolecular interactions result in deterioration of protective properties of CA.

The intercepts of the isotherms were then used to calculate the adsorption equilibrium constant (K_ads_). K_ads_ values can be used to calculate the free energy of adsorption (ΔG_ads_) according to the following equation [55]:

(14) $$∆G_{\mathrm{ads}}=-\mathrm{RT}\left(\ln K_{\mathrm{ads}}-\ln\frac{1}{55.5}\right)$$

where R denotes the universal gas constant, T is the absolute temperature and 55.5 is water solution concentration given in mol L^−1^. Calculated adsorption parameters are shown in Table 3.

Negative values of the free energy of adsorption can be attributed to the spontaneous adsorption of the CA on tested samples surfaces. Values of ΔG_ads_ of −20 kJ mol^−1^ or more indicate a physical nature of the adsorption process. On the other hand, ΔG_ads_ values of −40 kJ mol^−1^ or less can be ascribed to chemical adsorption [72]. Calculated values of ΔG_ads_ lay between −20 and −40 kJ mol^−1^ and thus it can be concluded that in the case of CA adsorption on Cu and brass sample surfaces occurs through a mixed physisorption–chemisorption mechanism [73]. However, in the case of Cu, participation of physisorption in the adsorption mechanism of CA is significantly higher than chemisorption due to its ΔG_ads_ value being close to −20 kJ mol^−1^. The calculated ΔG_ads_ value for Zn was −39.5 kJ mol^−1^ [50], indicating that although the adsorption still proceeds through the mixed physisorption–chemisorption mechanism, there is a considerable contribution of chemisorption in the process. Brass as an alloy composed of Zn and Cu is characterized by typical mixed mechanism of adsorption, considering that the ΔG_ads_ value is almost exactly in the middle of ΔG_ads_ range characteristic for physisorption–chemisorption. In the case of all tested samples, fitting the PDP data using the Langmuir isotherm suggests that their surfaces are covered with a monolayer of CA.

The impact of immersion time on the inhibitory properties of CA for Cu and brass was investigated in three consecutive measurements. Tafel plots obtained from PDP measurements are presented in Figures S1 and S2. Corrosion parameters obtained from these Tafel plots are shown in Table 4 and Table 5.

The inhibition efficiency of CA in three successive measurements was determined in regard to corrosion rates calculated in three consecutive measurements for samples immersed in 0.1 M NaCl without CA.

An increase in immersion time in NaCl resulted in a slight decrease in E_corr_ values for Cu and noticeable decrease (41 mV) for brass, indicating that the samples became more thermodynamically susceptible to corrosion. Additionally, a considerable increase (about 3.3 times) in CR of Cu can be observed with longer immersion times. For brass, on the other hand, the CR values did not change significantly throughout the three measurements.

Increasing exposure time to NaCl with optimal CA concentration resulted in a slight decrease in E_corr_ for both samples. Additionally, there was an increase in corrosion rate in the second measurement, which then became almost stable in the third measurement. The slight increase in inhibition efficiency after the third measurement in the case of Cu may be ascribed to the stabilization of the protective film formed on the tested sample surface, following the mixed physisorption–chemisorption mechanisms [56].

The CR of Cu immersed in NaCl with optimal concentration of CA resulted in a 2.4-fold increase. However, the comparison of CR values for samples immersed in NaCl and NaCl with the optimal concentration of CA revealed that the corresponding values of this parameter were significantly lower for samples immersed in the corrosive medium with the inhibitor, by a factor of at least 3.5. In the case of brass, the increase in CR for samples immersed in NaCl were noticeably less visible, as the difference between this parameter determined in the first and third measurement is 1.1-fold. The increase in CR for samples immersed in NaCl with CA was higher and differed from the first measurement by a factor of 1.8. Nevertheless, the corresponding CR values were noticeably lower for samples immersed in the tested corrosive medium with the added inhibitor, by a factor of at least 2.3.

Obtained results clearly show that CA can be applied as a green corrosion inhibitor for both Cu and brass, despite the fact that increasing immersion time in corrosive medium can result in a deterioration of the protective properties of the adsorbed inhibitor.

**Figure 5:**
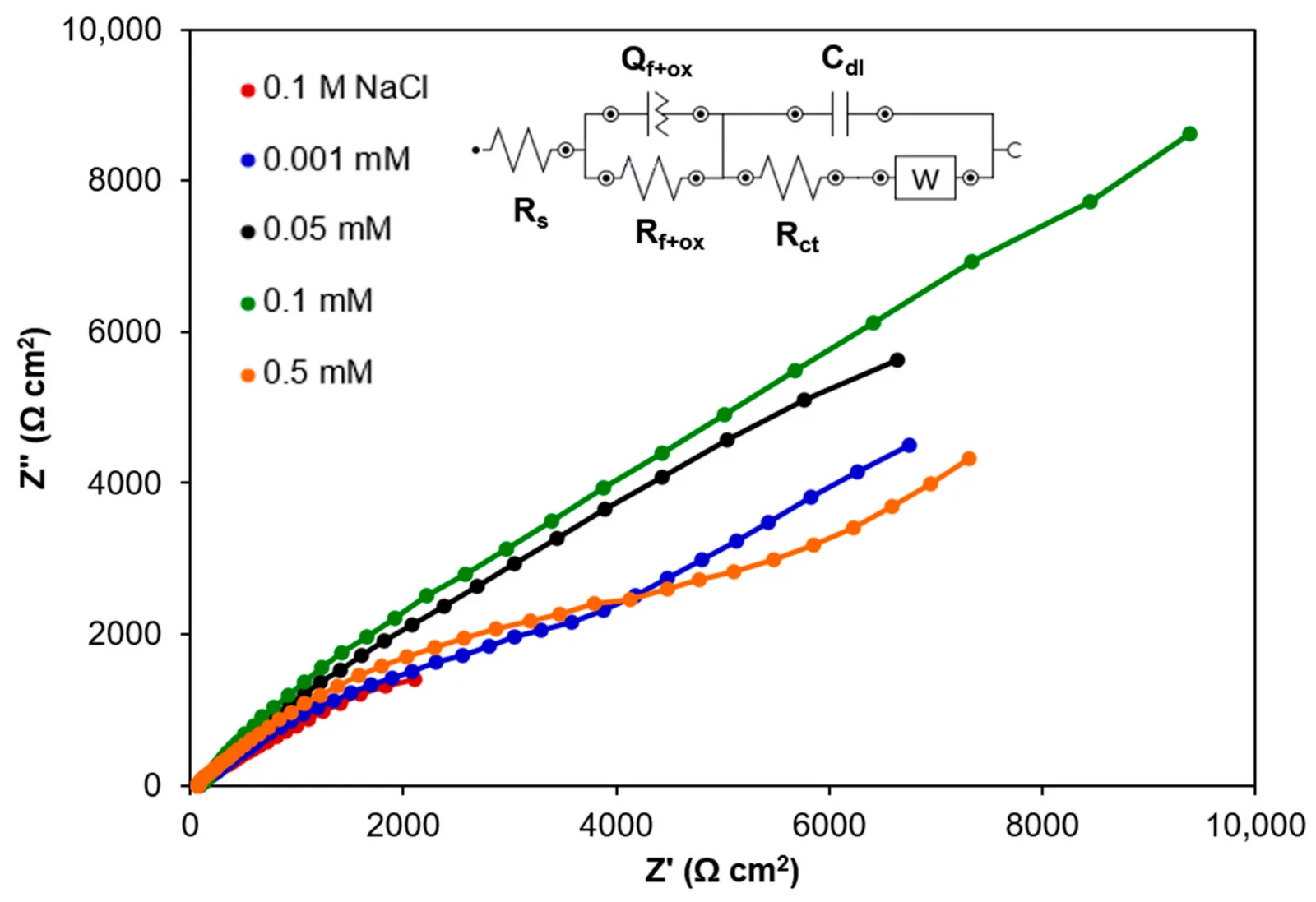
Exemplary Nyquist plots recorded for Cu samples in 0.1 M NaCl with and without the addition of CA. Inset: equivalent electrical circuit used for fitting the EIS data.

**Figure 6:**
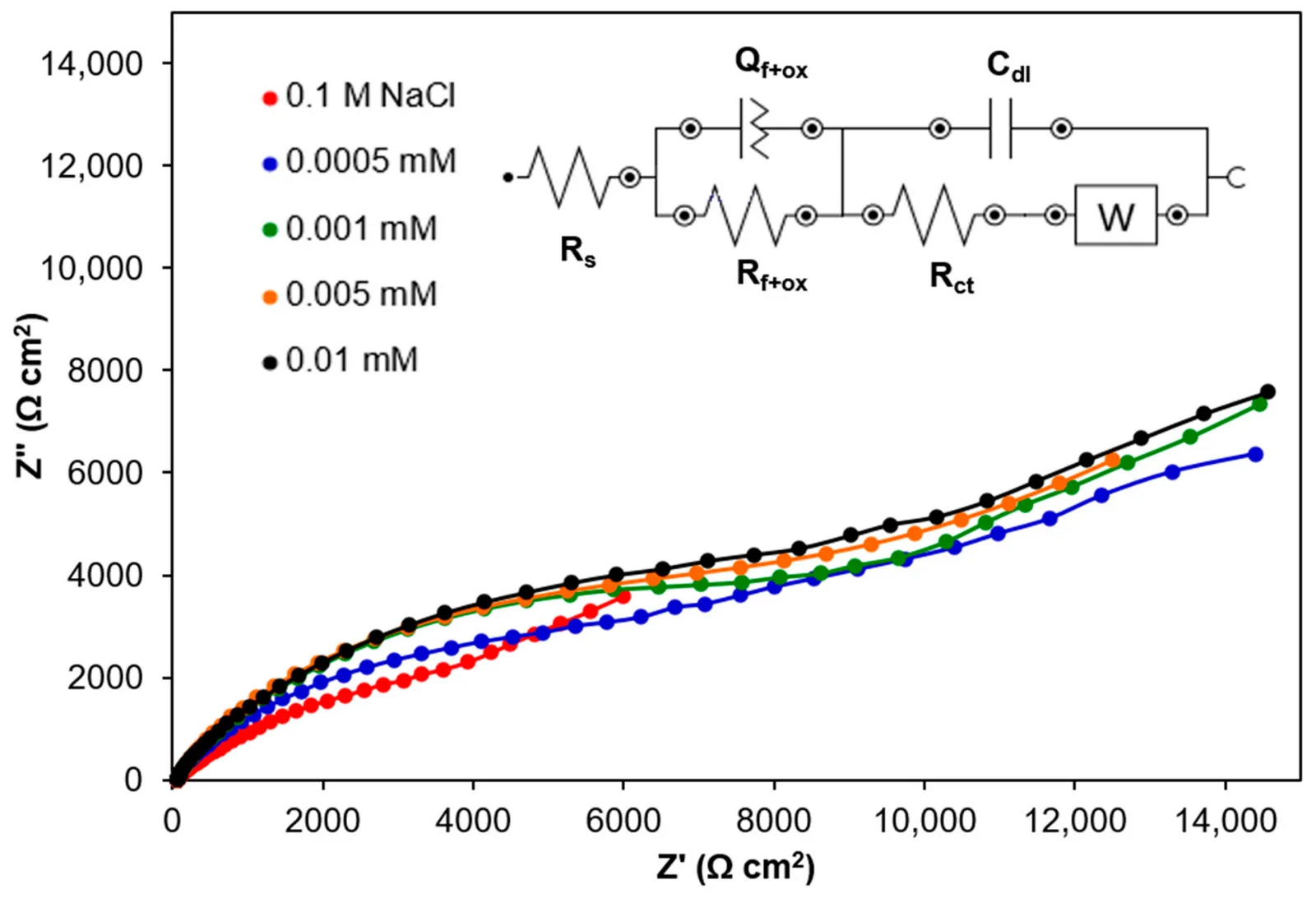
Exemplary Nyquist plots recorded for brass samples in 0.1 M NaCl with and without the addition of CA. Inset: equivalent electrical circuit used for fitting the EIS data.

In order to confirm the results obtained from PDP experiments, the EIS measurements were conducted. Exemplary Nyquist plots acquired from these tests are shown in Figure 5 (Cu) and Figure 6 (brass).

In order to explain the behavior of Cu and brass in NaCl in the presence of CA, the data obtained from the EIS experiments were fitted with the electrical equivalent circuits (EEC). The parameters characterizing elements of EEC were calculated and are presented in Table 6 and Table 7.

Nyquist plots recorded for Cu and brass in NaCl and with addition of CA (Figure 5 and Figure 6) at different concentrations are in the shape of a semicircle observed in the high-frequency region followed by a straight line in the low-frequency region. The semicircle can be ascribed to the charge transfer resistance of the electrical double layer, whereas the straight line reflects the diffusion-controlled process. Additionally, the shape of the Nyquist plots for samples tested in modified and unmodified corrosive medium remains almost the same, which indicates that there is no significant change in the electrochemical properties of the solution, apart from impedance [74]. The increase in the diameter of the capacitive loop can be attributed to the increase in charge transfer resistance and therefore higher inhibition efficiency. Nyquist plots presented in Figure 5 and Figure 6 can be fitted with the following equivalent electrical circuit: [R_s_(Q_f+ox_R_f+ox_)(C_dl_[R_ct_W])]. R_s_ denotes the solution resistance, Q_f+ox_ and R_f+ox_ are constant phase element and resistance of the inhibitor and corrosion products film formed on the substrate surfaces. C_dl_ represents the capacitance of the electrical double layer while R_ct_ is the charge transfer resistance, and W is the Warburg diffusion impedance. The circuit (Q_f+ox_R_f+ox_) with a constant phase element, replacing the ideal capacitor, and resistance can be attributed to the inhibitor film formed by its adsorption on the substrate surface. This circuit corresponds to high frequencies. At medium and lower frequencies, the second circuit (C_dl_[R_ct_W]) includes Warburg impedance, which corresponds to the semi-infinite linear diffusion through the substrate–electrolyte interface. The total impedance can be calculated according to the following equation:

(15) $$Z=R_{s}+\frac{1}{\frac{1}{R_{f+\mathrm{ox}}}+{\left(j\omega \right)}^{n_{f+\mathrm{ox}}}Q_{f+\mathrm{ox}}}+\frac{1}{+\frac{1}{R_{\mathrm{ct}}+Z_{W}}+j\omega C_{\mathrm{dl}}}$$

In the circuit (Q_f+ox_R_f+ox_), pure capacitance had to be replaced with constant phase element (Q) due to heterogeneity of the interface between the substrate surface covered with corrosion products, the adsorbed inhibitor, and NaCl solution. The parameter n is attributed to the surface irregularity. The Q impedance ($Z_{Q_{f+\mathrm{ox}}}$) can be calculated according to the following equation [75]:

(16) $$Z_{Q_{f+\mathrm{ox}}}=\frac{1}{Q_{f+\mathrm{ox}}{\left(j\omega \right)}^{n_{f+\mathrm{ox}}}}$$

Warburg impedance used in the second circuit (C_dl_[R_ct_W]) can be calculated using the following equation [76,77]:

(17) $$Z_{W}=\frac{\sigma \left(1-j\right)}{\sqrt{j\omega }}$$

where σ is the Warburg coefficient. The inhibition efficiency (IE) of the inhibitor was evaluated on the basis of the total polarization resistance (R_p_) calculated according to the following equation:

R_p_ = R_ct_ + R_f+ox_ (18)

The chi-square parameter was used to evaluate the goodness of fit of electrical equivalent circuit to obtained EIS data. Its values were in the range of 0.003–0.052, proving good fitting of the circuits.

The alternative circuit [R([R(Q[RW])]C)] was also evaluated but provides significantly less satisfactory description of the experimental data. The selection of the former circuit was supported by its better agreement with the measured impedance spectra, e.g., lower chi-squared values and lower estimated fitting errors for the individual circuit elements. Using the same equivalent circuit for both blank NaCl- and CA-containing solutions also enabled a consistent comparison of the fitted parameters and the changes in electrochemical response associated with the addition of the inhibitor.

In the high-frequency region, the diameter of the semicircles visible in the Nyquist plots increases with an increase in CA concentration. However, at CA concentrations higher than 0.1 mM for Cu and 0.01 mM for brass, a decrease in the semicircle diameter is observed, resulting in lower values of charge transfer resistance and a higher corrosion rate. This confirms that the CA concentration of 0.1 mM and 0.01 mM for Cu and brass, respectively, is optimal and effectively prevents the charge transfer reaction, resulting in a decrease in the corrosion rate (Table 6 and Table 7).

The values of parameters characterizing the EEC listed in Table 6 show that in the case of Cu, the capacity of the electric double layer (C_dl_) decreased and achieved the lowest value at CA concentration of 0.1 mM. This means that increasing CA concentration results in higher adsorption of CA and formation of a protective layer. Simultaneously, the R_ct_ value increased from 536 Ω in blank solution to 1165 Ω in 0.1 mM CA, indicating the significant reduction in the corrosion rate. The increase in the R_f+ox_ value probably results from increasing film thickness on Cu (Table 6). The parameter n_f+ox_ is almost constant and equal to 0.45, indicating high metal surface roughness [78]. Furthermore, n_f+ox_ values close to 0.5 imply Warburg-type behavior and diffusion effects [79]. The brass samples are characterized by higher n_f+ox_ values (Table 7), proving less surface roughness, resulting in higher R_f+ox_ values in comparison to Cu.

The Warburg impedance increasing with the increase in CA concentration shows that at medium and lower frequencies, the diffusion processes play a significant role in the corrosion phenomena. In the case of brass, the Warburg impedance lowers in the presence of CA, and this proves that the effect of diffusion is clearly weakened [4]. Simultaneously, the lowering values of C_dl_ indicate an increase in thickness of the electric double layer due to higher adsorption of CA.

#### 2.1.2. Corrosion Inhibition by CA Coatings

Taking into consideration the possibility of applying CA as green corrosion inhibitor for historic heritage artefacts, CA coatings were deposited on Cu and brass sample surfaces. Different concentrations of CA solutions in ethanol were prepared. Such solutions were then brushed onto the sample surface several times, forming different numbers of layers. The concentration of CA solutions was in the range of 5 to 30 mM. Each layer was dried in warm air to ensure the evaporation of ethanol.

**Figure 7:**
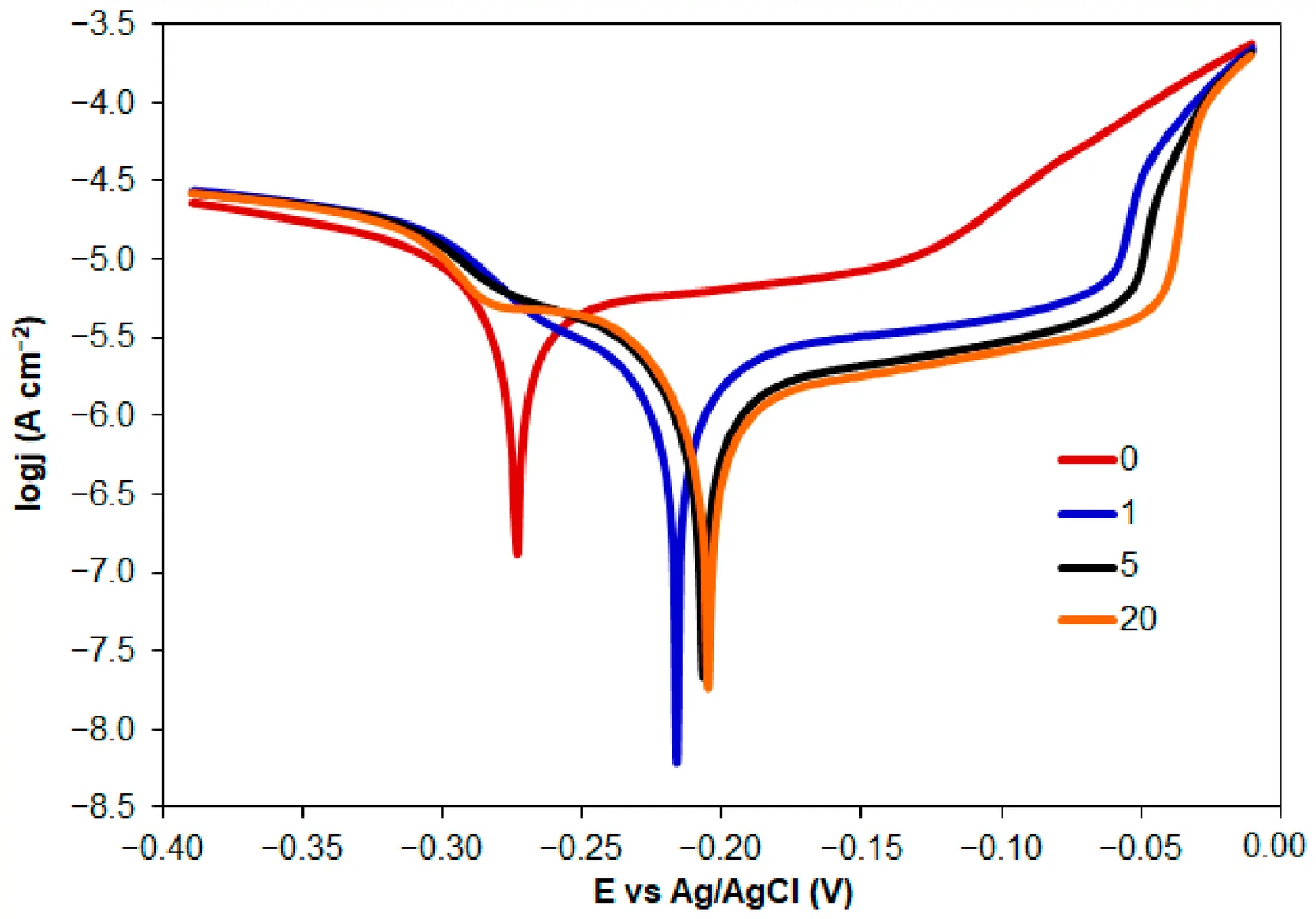
Exemplary Tafel plots recorded for Cu coated with various number of layers formed from 5 mM CA solution.

**Figure 8:**
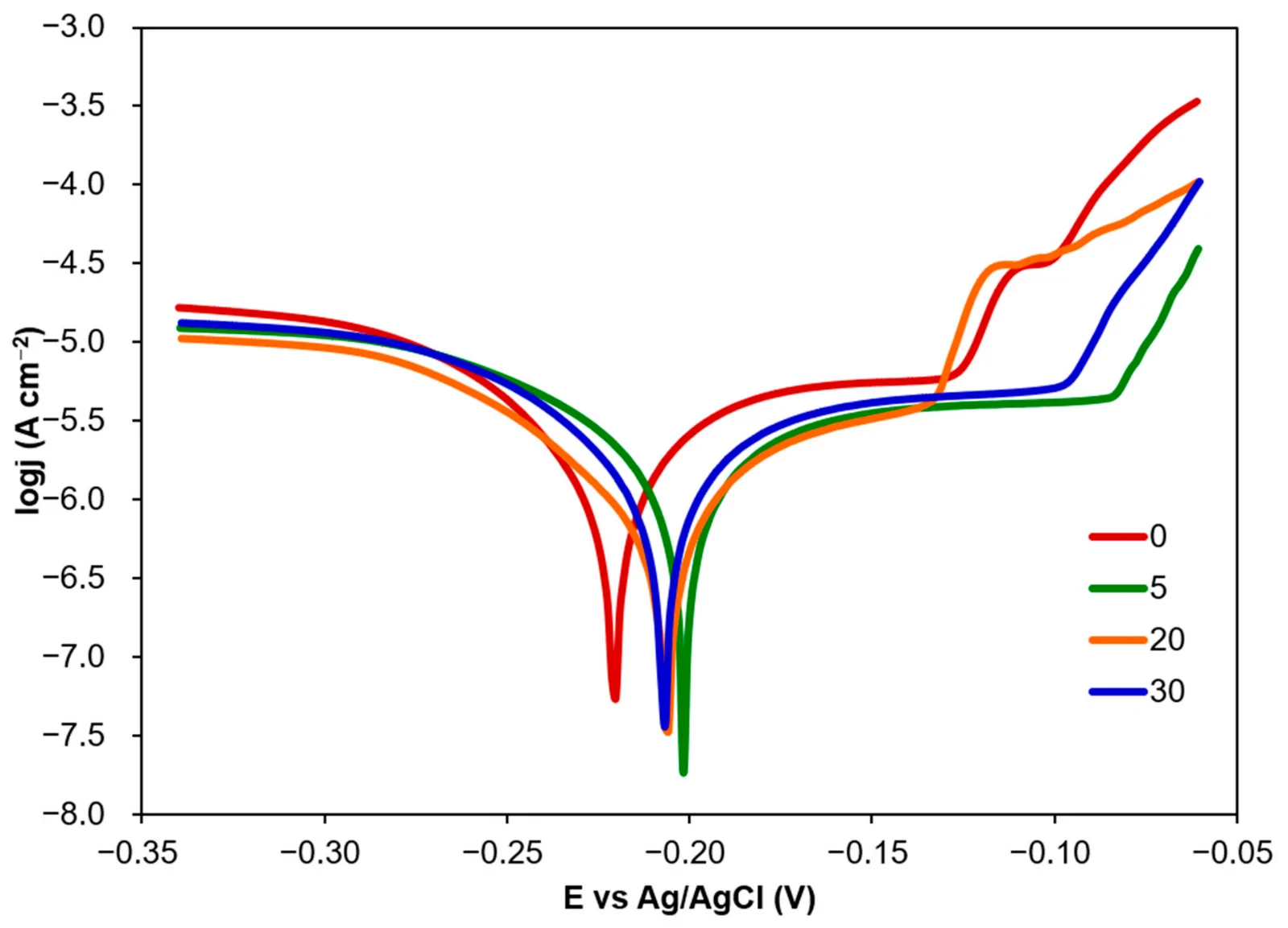
Exemplary Tafel plots recorded for brass coated with different number of layers formed from 10 mM CA solution.

Similarly to the corrosive medium modification studies, PDP curves were recorded for pure Cu and brass with different number of layers formed from solutions of various concentrations. Investigations were performed in 0.1 M NaCl also in three consecutive experiments with a 1 h OCP measurement taken before them. Exemplary Tafel plots are presented in Figure 7 and Figure 8 and corrosion parameters calculated for Cu and brass are shown in Table 8 and Table 9. Additionally, the pseudo-porosity of CA coatings on Cu and brass was calculated according to the following equation [80,81,82,83]:

(19) $$P=\frac{R_{p_{s}}}{R_{p}}\cdot 10^{-\left|\frac{∆E_{\mathrm{corr}}}{b_{a}}\right|}\cdot 100$$

where R_ps_ and R_p_ are the polarization resistances of the pure sample and sample with coating, respectively, ΔE_corr_ is the difference between corrosion potential of the pure sample and sample with coating and b_a_ is the anodic Tafel slope for the sample without coating.

CA coatings applied on Cu surface resulted in a noticeable shift of E_corr_ towards more positive values but not higher than 85 mV in comparison to the pure Cu sample. This means that CA coatings slow down both anodic and cathodic reactions. Additionally, a clear decrease in b_a_ values indicates that CA coatings retard mainly the anodic dissolution of Cu. Furthermore, regardless of the number of layers and the CA solution concentration, the IE values were higher than 70%. However, the highest IE value (87%) was obtained for 20 layers formed from 5 mM CA solution. The increase in the number of layers up to 30 caused a slight decrease in the IE value due to the possible deterioration of protective properties of the coating. It was confirmed by calculation of the pseudo-porosity value, which increased.

In the case of brass, changes in E_corr_ values of samples with and without CA coating were very slight. Additionally, b_a_ and b_c_ values were slightly lower for coated brass samples, indicating that CA coatings retard both anodic and cathodic reactions. However, IE values were lower in comparison to Cu and Zn [50]. The highest IE value (81%) was achieved for 20 layers formed from 10 mM CA solution. A higher number of layers (30) resulted in a deterioration of IE and a simultaneous increase in pseudo-porosity, probably due to less uniform coating and its weaker adhesion to the substrate surface. Furthermore, changes in b_a_ and b_c_ values for pure and coated brass samples were significantly lower than in the case of Cu, indicating the lack of change in corrosion mechanism. Applying coatings obtained from solutions of higher CA concentration (20 mM) resulted in noticeably higher pseudo-porosity and lower IE values. In general, pseudo-porosity calculated for brass was clearly higher than in the case of Cu coatings. This can probably be attributed to the dezincification of brass with consequent formation of a porous Cu-rich layer.

Similarly to the case of medium modification, the effect of immersion time on protective properties of CA coatings was investigated in three consecutive measurements. Results are presented in Figures S3 and S4, and the corresponding corrosion parameters are presented in Table 10 and Table 11.

In the case of both materials tests of the impact of immersion time on CA coatings revealed that they are more stable in comparison to Zn [50]. The highest inhibition efficiency for Cu (91.7%) was obtained after 2 h of immersion and remained nearly constant after 3 h. For brass, the best results were also obtained for 2 h of immersion time with an inhibition efficiency of 81.7%. Simultaneously, lower pseudo-porosity was observed. Stabilization of CA coatings protective properties can be explained by formation of corrosion products which are deposited on CA coating making it more uniform and less porous.

For Cu, the PDP curve recorded in the third measurement (Figure S3) is different from the curves obtained from the first and second measurements. The first peak observed at −0.284 V was used in comparison of E_corr_ values determined in three consecutive measurements. The second (−0.272 V) and third (−0.220 V) E_corr_ can be ascribed to active–passive transition region of Cu resulting from the formation of different corrosion products [84,85] proved by TOF-SIMS measurements. Similar active–passive regions were observed in the case of Tafel plots recorded for the second brass component (Zn) samples [50].

**Figure 9:**
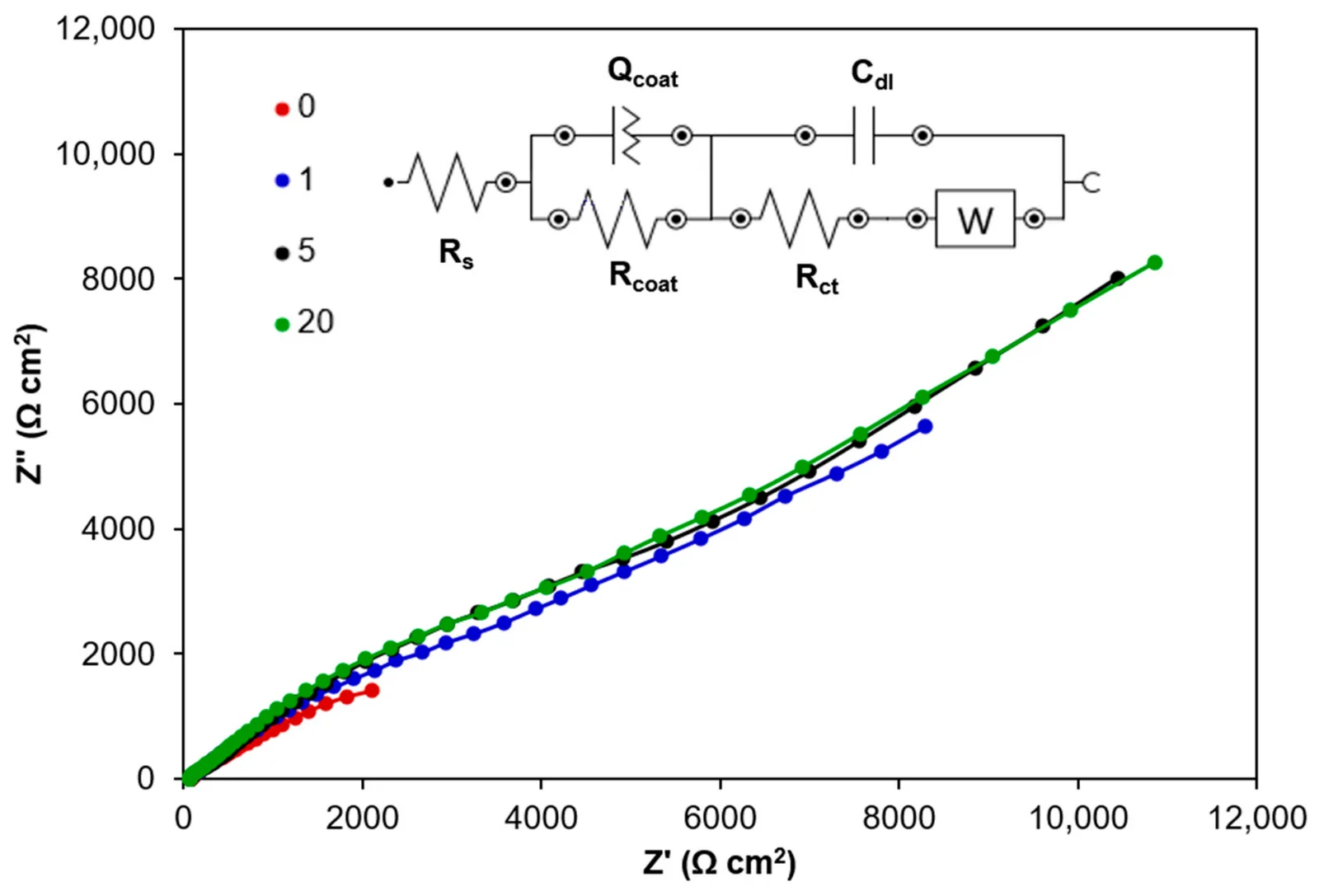
Exemplary Nyquist plots obtained for Cu samples in 0.1 M NaCl with various numbers of layers formed from 5 mM CA solution.

**Figure 10:**
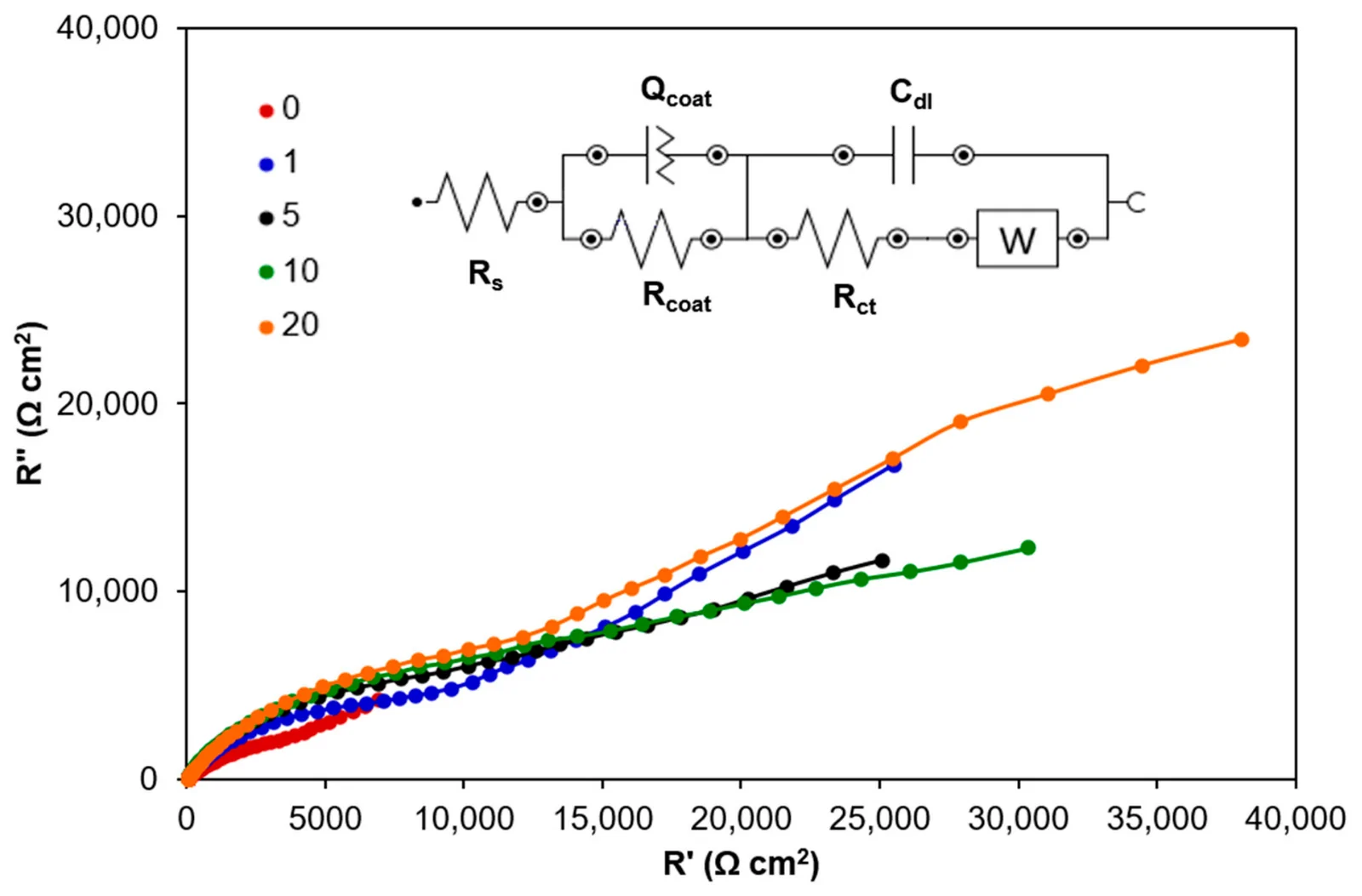
Exemplary Nyquist plots obtained for brass samples in 0.1 M NaCl with various numbers of layers formed from 10 mM CA solution.

In order to confirm PDP results for the coated samples and further investigate the properties of these coatings, EIS measurements were performed. EIS experiments were conducted for the optimal concentration of CA determined in the PDP measurements. Exemplary Nyquist plots for Cu coated with different numbers of layers formed from 5 mM CA solution (Figure 9) and brass coated with different numbers of layers formed form 10 mM CA solution (Figure 10) are presented.

The corrosion behavior of Cu and brass coated with CA was evaluated based on the data obtained from the EIS experiments fitted with the electrical equivalent circuits (EECs). The parameters characterizing elements of the EECs were calculated and are presented in Table 12 and Table 13.

Similarly to corrosive medium modification, in all Nyquist plots recorded for samples coated with CA, a semicircle in the higher frequency region followed by a straight line in the lower frequency region can be observed. Both in the case of Cu and brass, the EIS data can be fitted by the following equivalent circuit: [R_s_(R_coat_Q_coat_)(C_dl_[R_ct_W])]. The (R_coat_Q_coat_) circuit can be attributed to the CA coatings on the substrates surfaces, while the (C_dl_[R_ct_W]) circuit also includes Warburg impedance, which corresponds to the semi-infinite linear diffusion through the substrate–electrolyte interface. In the case of samples without CA coatings, R_coat_ and Q_coat_ correspond to R_ox_ and Q_ox_. The increase in the diameter of the semicircle for samples with CA coatings indicates increasing corrosion resistance. The inhibition efficiency was evaluated based on polarization resistance (R_p_), defined by the sum of R_coat_ and R_ct_.

In the case of both substrates, depositing a higher number of CA layers resulted in an increase in R_coat_ values, indicating higher resistance of the protected samples to corrosion. However, in the case of Cu, depositing more than 20 layers caused a decrease in R_p_ values, probably due to formation of less uniform and dense coating. The highest IE value was obtained for 20 layers and was equal to 77%. For brass, the increase in number of layers also resulted in the higher values of R_coat_ but for more than 20 layers R_coat_ was almost constant. The highest IE value was observed for 20 and 30 layers and was equal to 71%. These results confirmed the results of PDP experiments.

Data related to the EEC and presented in Table 12 show that in the case of Cu, Q_coat_ values representing the capacity of the coating on the substrate clearly decrease in comparison to pure substrate. Moreover, the higher the number of layers in the coating, the lower the value of Q_coat_, except for the highest number of layers (30). Simultaneously, values of n_coat_ parameter related to Q_coat_ element increased to about 0.7 for coatings in comparison to pure substrate (n = 0.46), and this indicates an increase in the homogeneity and uniformity of the surfaces with the coating [25]. Furthermore, a higher number of layers in the coating results in a higher R_coat_ value and higher resistance to corrosion. The Warburg element in the EEC proves that diffusion processes play a significant role in corrosion phenomena for Cu with coatings as well as for pure substrate.

In the case of brass, Q_coat_ values (Table 13) are also lower in comparison to pure substrate, but the n_coat_ parameter is characterized by slightly higher values, indicating slightly higher homogeneity and uniformity of the coating in comparison to the coated Cu samples. At the same time, the higher the number of layers in the coating, the higher the R_coat_ value and the higher the resistance to corrosion. The Warburg impedance values are lower for brass with the coating in comparison to the pure substrate, proving that the coating weakens the effect of diffusion processes on corrosion rate.

### 2.2. Morphological Characterization of Cu and Brass Surfaces

Scanning electron microscopy (SEM) coupled with energy-dispersive X-ray spectroscopy (EDS) was used to examine the surface morphology of Cu and brass samples with and without coatings formed from CA solutions, before and after immersion in NaCl solution.

**Figure 11:**
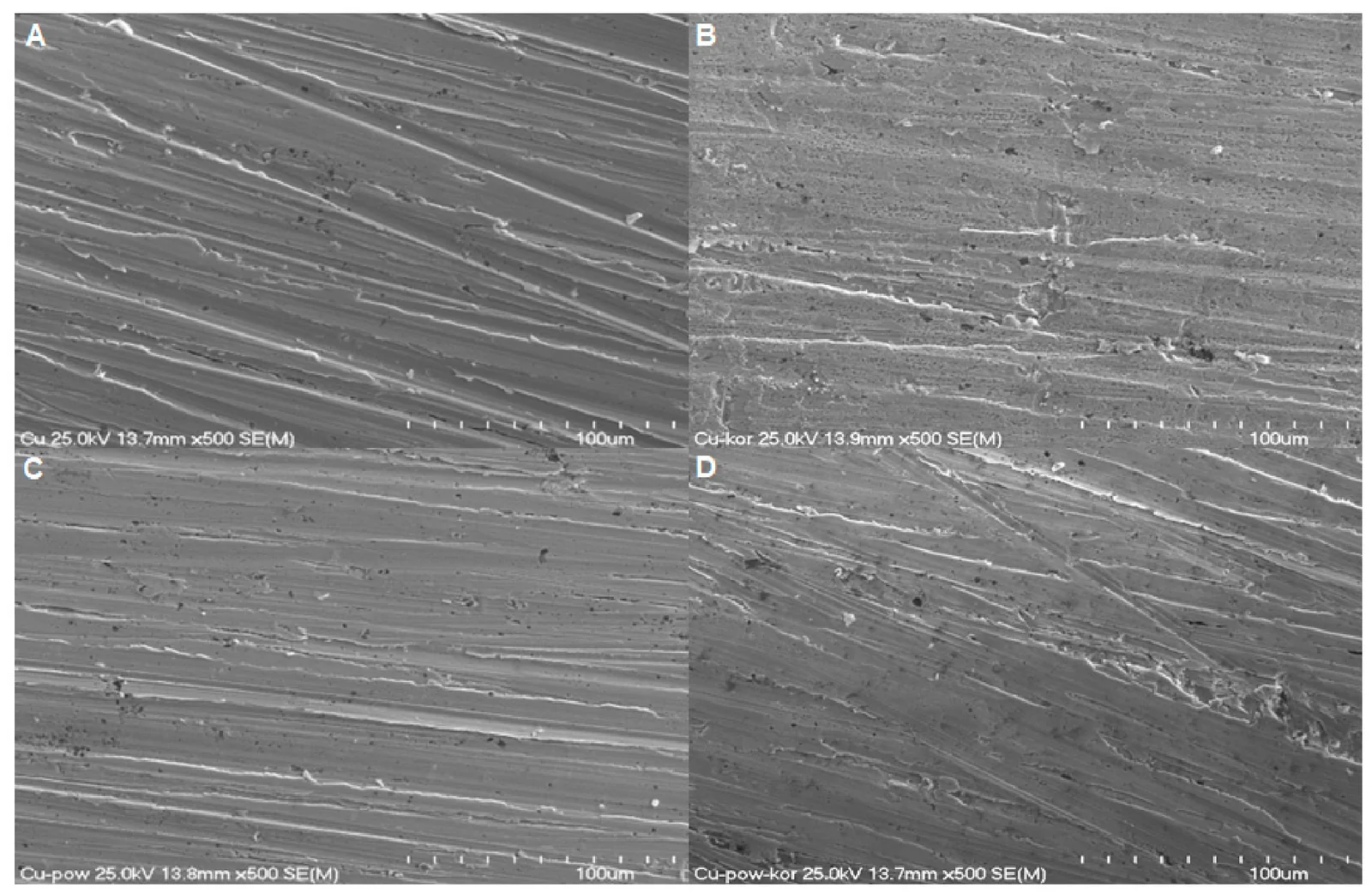
SEM images of uncoated Cu (**A**), Cu after immersion in 0.1 M NaCl (**B**), Cu with 20 layers of CA formed from 10 mM solution (**C**) and Cu with 20 layers after immersion in 0.1 M NaCl (**D**).

**Figure 12:**
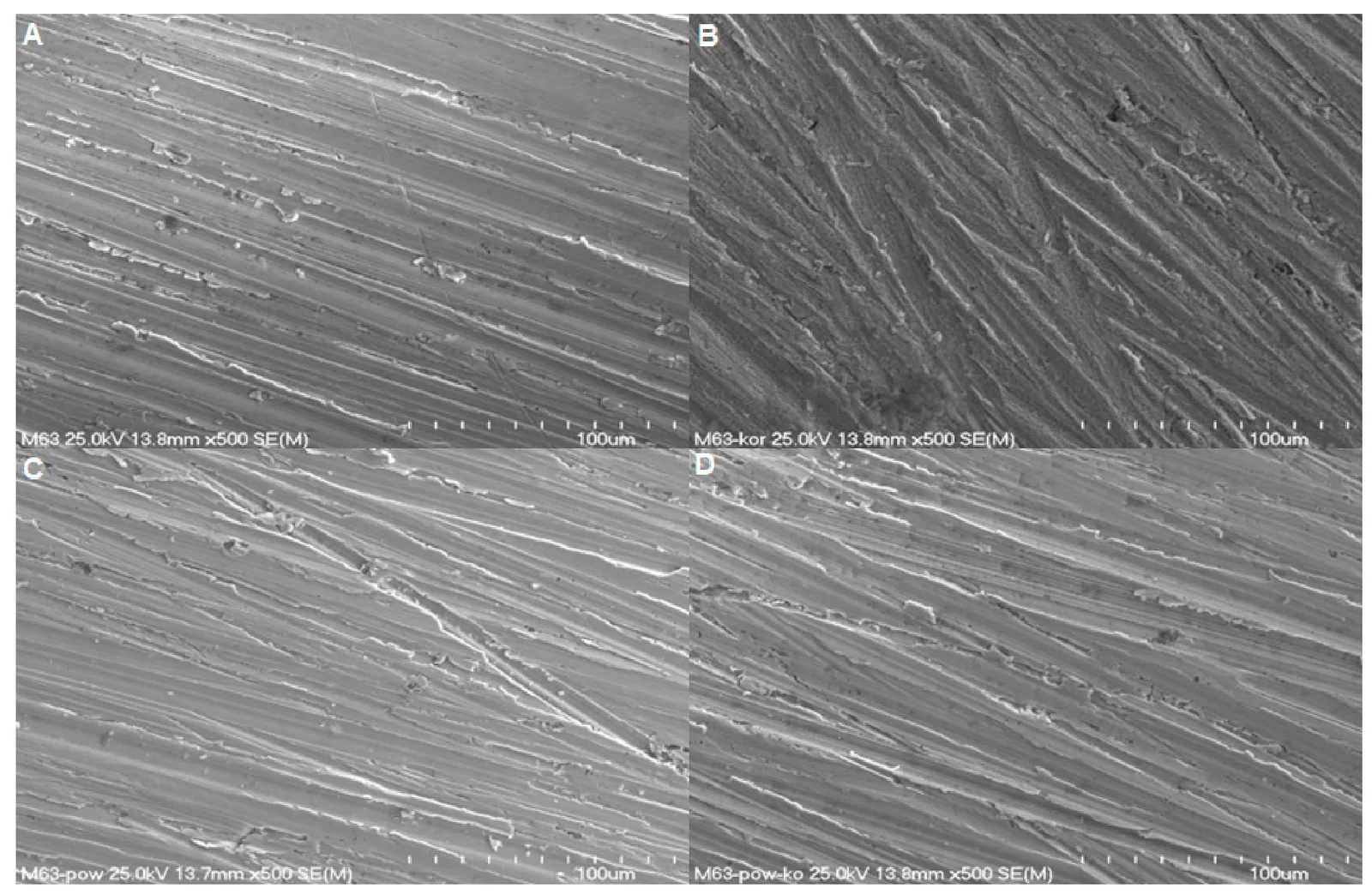
SEM images of uncoated brass (**A**), brass after immersion in 0.1 M NaCl (**B**), brass with 20 layers of CA formed from 20 mM solution (**C**) and brass with 20 layers after immersion in 0.1 M NaCl (**D**).

In the case of the uncoated samples, characteristic grinding and polishing scratches resulting from the sample preparation procedure can be observed. After immersing the uncoated samples in 0.1 M NaCl, surface features consistent with the formation of corrosion products can be noted (Figure 11B and Figure 12B). The surfaces of the samples coated with CA protective layers are noticeably more uniform, with significantly fewer scratches visible. After immersion in 0.1 M NaCl, the surface of Cu coated with 20 layers of CA (Figure 11D), appears less porous compared with the uncoated specimen, which may be related to a lower extent of corrosion products formed on the surface. In the case of brass coated with protective layers, the surface after immersion in the corrosive medium (Figure 12D) is clearly more uniform, and only a limited amount of corrosion products is visible.

Furthermore, EDS elemental maps of carbon were acquired to provide indirect evidence for the presence of caffeic acid coating on Cu and brass surfaces (Figure S5).

In the case of surfaces coated with CA, a higher carbon content is observed, which is consistent with the chemical composition of caffeic acid. Coatings seem to be almost uniformly distributed on the Cu and brass surfaces. Small amounts of carbon detected on the uncoated samples may be attributed to surface contamination and/or the carbon-based mounting material used during SEM analysis.

The spectra of EDS recorded for uncoated samples of Cu and brass before and after immersion in 0.1 M NaCl solution as well as for Cu and brass coated with CA before and after exposure to the same corrosive medium, are presented in Figures S6 and S7. The corresponding elemental compositions obtained from EDS spectra are shown in Table 14 and Table 15.

In the EDS spectra, the most abundant elements are C, O, Cu and Zn, the last one being detected only in the case of brass. The simultaneous detection of Cu and O is consistent with the presence of copper-containing corrosion products, likely including oxides, while the simultaneous presence of Zn and O in brass may indicate the formation of zinc oxide corrosion products, as described by Reactions (2)–(11).

The elemental composition of the uncoated Cu surface consists of approximately 89% Cu with a small content of C (9.5%) most likely coming from the atmospheric contamination and/or carbon tape. After exposure to NaCl solution, the Cu and C contents remain relatively similar whereas the O content increases from 0.5% to 4.7%, suggesting formation of copper oxide corrosion products. In the case of Cu coated with CA layers, the Cu content remains nearly constant both before and after exposure to NaCl. However, the C and O contents are higher than those observed for the unprotected samples and are consistent with the presence of CA layers on the surface. After immersion in NaCl solution, the content of C and O is nearly constant, suggesting a reduced extent of corrosion product formation compared with the uncoated sample. These results support the protective effect of CA coating.

Brass samples contain Zn and Cu, which is in agreement with the information provided by the manufacturer. In the case of unprotected samples, a noticeable increase in the O content to 6.6% after exposure to NaCl solution is observed (Table 15). This can be attributed to the formation of oxide corrosion products of both major components of brass. Coating the brass surface with CA results in higher contents of C and O in comparison to the unprotected sample (Table 15). This is consistent with the presence of CA on the brass surface. After immersion in NaCl solution, the elemental composition does not change significantly, with the exception of the O content, which is slightly higher (2.7%). This may be attributed to the formation of Cu- and Zn-containing oxide corrosion products. Nevertheless, the oxygen content remains lower than that observed for the unprotected sample.

The detected Al signals most likely originate from the sample holder, polishing residues, or instrumental/background contributions rather than from the investigated materials themselves.

Brass samples exhibit the content of Zn and Cu which is in agreement with the information provided by the manufacturer. In the case of unprotected samples, a noticeable increase in O content to 6.6% after exposure to NaCl is observed (Table 15). This can be attributed to the formation of oxide corrosion products of both components of brass. Coating the brass surface with CA results in higher content of C and O in comparison to the unprotected sample (Table 15). This confirms the presence of CA on the brass surface. After immersion in NaCl, the elemental composition does not change significantly, with the exception of O content, which is slightly higher (2.7%). This indicates the formation of Cu and Zn oxide corrosion products. Nevertheless, their contents are lower than in the case of the unprotected sample.

**Figure 13:**
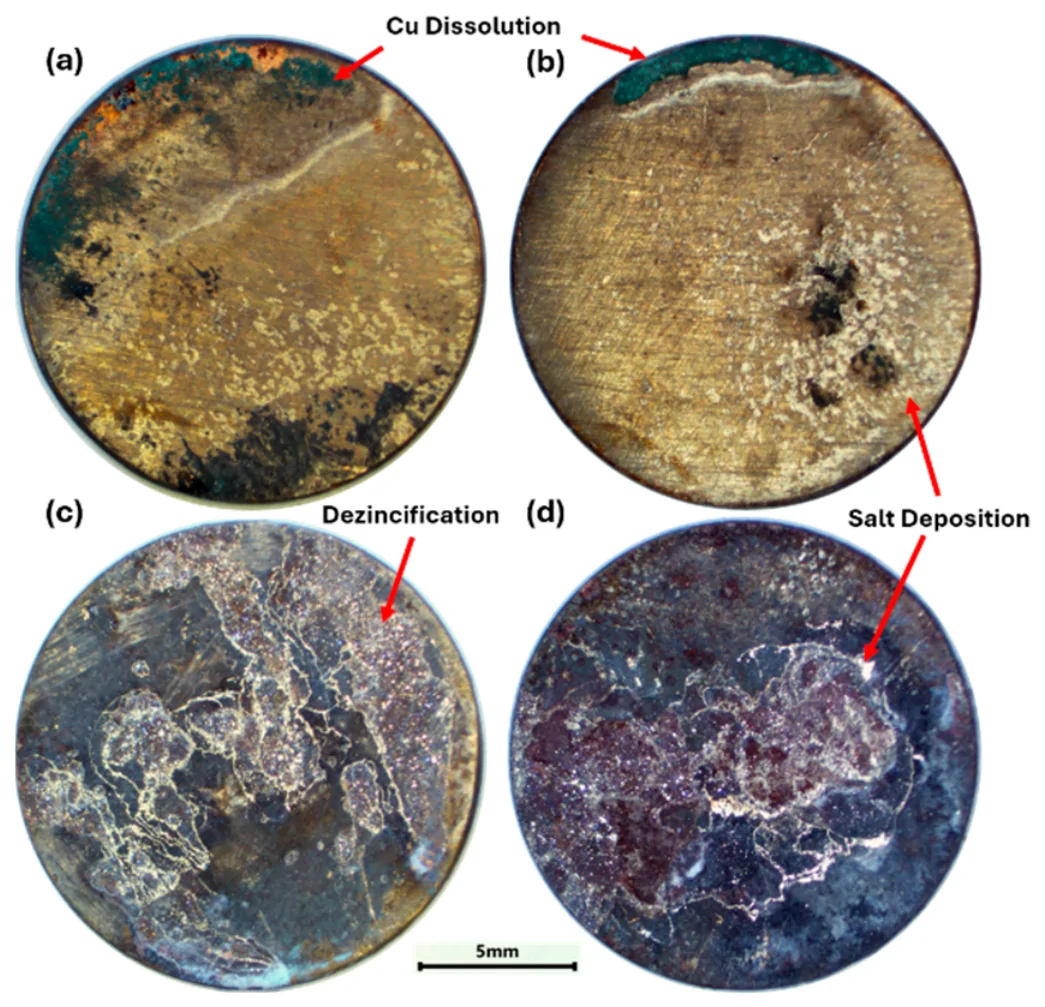
Stereomicroscopic images of (**a**) bare Cu, (**b**) 20 layers CA-coated Cu, (**c**) bare brass, and (**d**) 20 layers CA-coated brass samples after exposure to 5 wt.% NaCl at 35 °C for 7 days.

In order to further investigate the protective properties of CA coatings, the corrosion resistance of bare Cu and Cu coated with 20 layers of CA as well as bare brass and brass coated with 20 layers of CA was evaluated after 7 days (168 h) of exposure to a 5 wt.% NaCl salt spray environment at 35 °C. The selected environment provides an aggressive chloride-containing atmosphere capable of accelerating the electrochemical processes responsible for copper degradation and therefore provides a useful comparative assessment of the barrier performance of the CA coating. Exemplary optical micrographs of the samples with and without CA coatings are presented in Figure 13b,d and Figure 13a,c, respectively.

As shown in Figure 13a–d, the optical micrographs provide clear visual evidence of the protective effect of the 20-layer CA coating. Bare Cu (Figure 13a) exhibits extensive surface dissolution and severe corrosion product accumulation, indicating direct exposure of the substrate to the chloride-containing environment. In contrast, CA-coated Cu (Figure 13b) shows substantially less overall degradation, with localized Cu dissolution concentrated at the region where the coating was mechanically damaged, demonstrating that corrosion is strongly associated with a loss of coating integrity. For brass, Figure 13c shows pronounced dezincification accompanied by substantial salt/corrosion product deposition, consistent with the heterogeneous Cu/Zn and Cl distributions observed by EDS and indicating chloride-assisted preferential Zn dissolution. The 20-layer CA-coated brass (Figure 13d) exhibits significantly reduced surface damage and less pronounced dezincification, together with reduced chloride-associated deposits. Collectively, the optical and EDS results (discussed below) demonstrate that the multilayer CA coating acts as an effective barrier to electrolyte and chloride transport, suppressing localized Cu dissolution and, more importantly, reducing the preferential Zn dissolution responsible for dezincification of brass.

**Figure 14:**
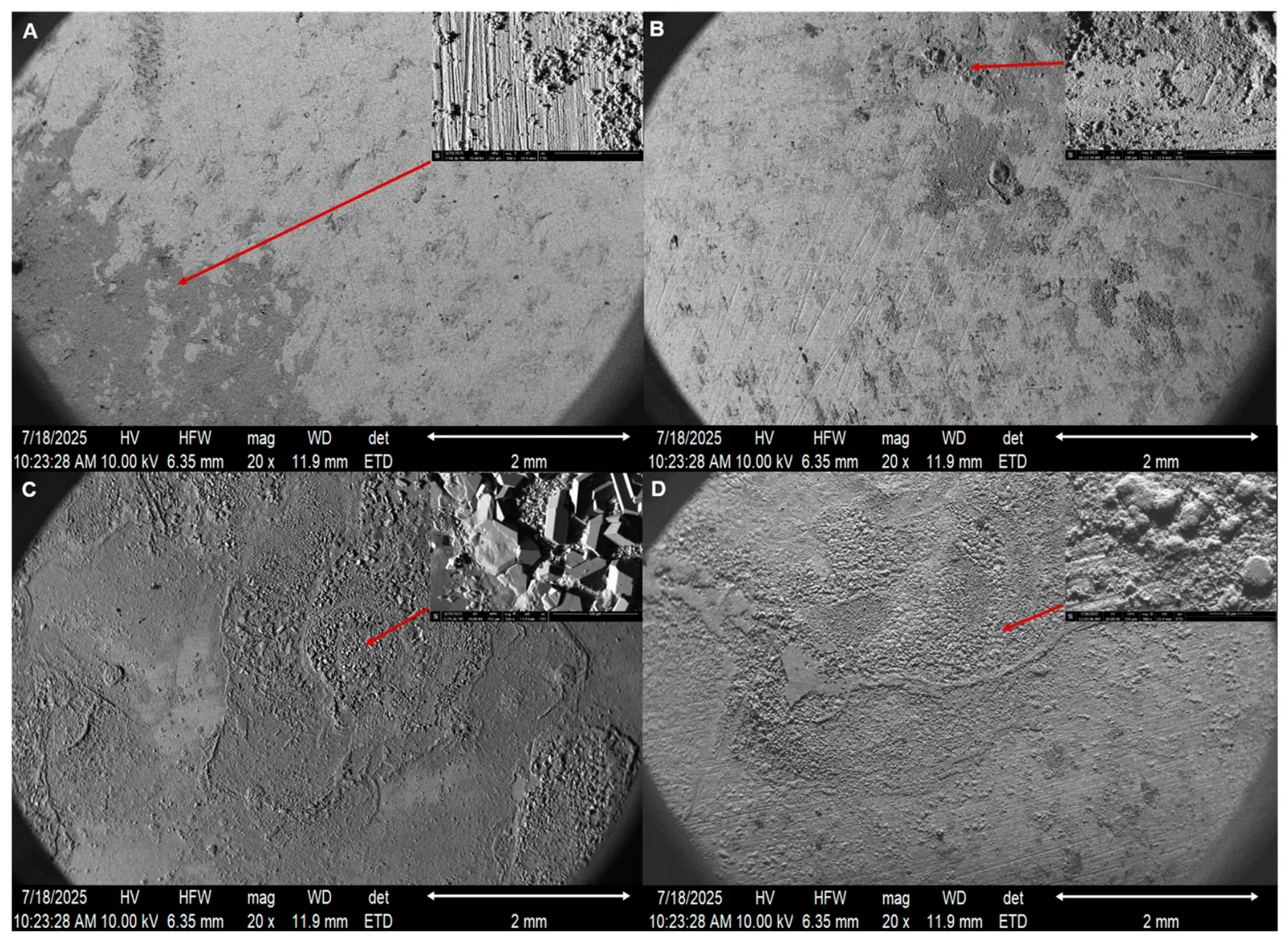
SEM images of Cu after a week-long exposure to NaCl in a salt spray chamber (**A**), Cu coated with 20 layers after a week-long exposure to NaCl in a salt spray chamber (**B**), brass after a week-long exposure to NaCl in a salt spray chamber (**C**) and brass coated with 20 layers after a week-long exposure to NaCl in a salt spray chamber (**D**).

In order to investigate the differences in surface morphologies, SEM-EDS experiments were performed for samples exposed to a salt spray environment. Their results are presented in Figure 14, Figure S8 and S9.

The SEM–EDS results in Figure 14 demonstrate a pronounced difference between the uncoated and CA-coated Cu surfaces, highlighting the effectiveness of the multilayer architecture in restricting chloride-induced degradation.

The SEM image of uncoated Cu (Figure 14A) reveals a substantially heterogeneous surface after 7 days, characterized by irregular deposits, agglomerated corrosion products, and localized surface attack. The corresponding EDS maps (Figure S8A) show a spatially heterogeneous Cu distribution accompanied by pronounced Cl-rich regions, particularly around surface deposits. This behavior is consistent with the established susceptibility of Cu to chloride-assisted corrosion. Chloride ions can participate in Cu dissolution and the formation of soluble and/or poorly protective Cu–Cl intermediates, followed by hydrolysis and oxidation reactions that generate complex Cu(I)/Cu(II) corrosion products [86]. The localized Cl enrichment further indicates that corrosion is spatially heterogeneous, with chloride accumulation contributing to the development of localized electrochemical sites. Under atmospheric conditions, such processes occur within thin electrolyte layers generated by condensation or salt deliquescence. Increasing humidity enhances electrolyte film persistence and electrochemical activity, while chloride further accelerates both anodic and cathodic reactions [87].

In contrast, the 20-layer CA-coated Cu (Figure 14B) exhibits substantially less pronounced surface degradation. The comparatively continuous morphology and reduced occurrence of localized corrosion features indicate that the multilayer coating effectively separates the Cu substrate from the corrosive environment. The EDS maps (Figure S8B) further show that Cu remains predominantly associated with the underlying substrate, whereas Cl is mainly distributed within or over the coating region rather than exhibiting the intense, localized Cu–Cl association observed for bare Cu. This spatial separation provides direct evidence of restricted electrolyte transport through the multilayer structure. The protection is attributed primarily to the decreased ionic transport through the 20-layer architecture, which increases the effective pathway for water, dissolved oxygen, and Cl^–^ to reach the metal/coating interface [88]. Although the presence of Cl^–^ within the coated region indicates that the CA layer is not completely impermeable, the substantially reduced localization of chloride at the Cu surface suggests that the coating acts as a kinetically effective diffusion barrier.

Under similar conditions of exposure, a different corrosion mechanism is observed for brass. The uncoated brass surface (Figure 14C) exhibits severe morphological degradation, localized attack, and regions characteristic of a porous or dealloyed structure. Unlike pure Cu, brass contains Zn, introducing the possibility of selective dissolution and dezincification. The Cu and Zn EDS maps (Figure S9A) reveal substantial compositional heterogeneity, while pronounced Cl^–^ accumulation occurs across attacked regions. Such behavior is consistent with preferential Zn dissolution, which can leave behind a porous Cu-enriched layer and progressively compromise the integrity of the alloy [89]. Chloride-assisted dissolution and complexation can further sustain metal-ion transport and promote coupled processes of Zn dissolution, Cu dissolution, and possible Cu redeposition [90]. Thus, the corrosion of brass involves not only surface oxidation but also microstructural dealloying, making the resulting damage potentially more detrimental than conventional surface corrosion of pure Cu.

The 20-layer CA-coated brass exhibits a significantly different response (Figure 14D and Figure S9B). Its comparatively continuous morphology, more homogeneous Cu/Zn distributions, and substantially weaker and more localized Cl^–^ signal indicate effective restriction of saline electrolyte ingress. Importantly, the preservation of a relatively uniform Zn distribution suggests suppression of the preferential Zn dissolution responsible for dezincification. The CA multilayer therefore provides protection against both conventional chloride-induced corrosion and the alloy-specific dealloying mechanism. Overall, these results demonstrate that multilayer CA protection functions as an effective diffusion barrier: in Cu, it suppresses localized chloride-assisted corrosion, whereas in brass it additionally limits Zn dissolution and the development of a porous Cu-rich dezincified layer. This dual protective effect highlights the potential of multilayer CA architectures for extending the durability of Cu-based materials under humid and chloride-contaminated atmospheric conditions.

**Figure 15:**
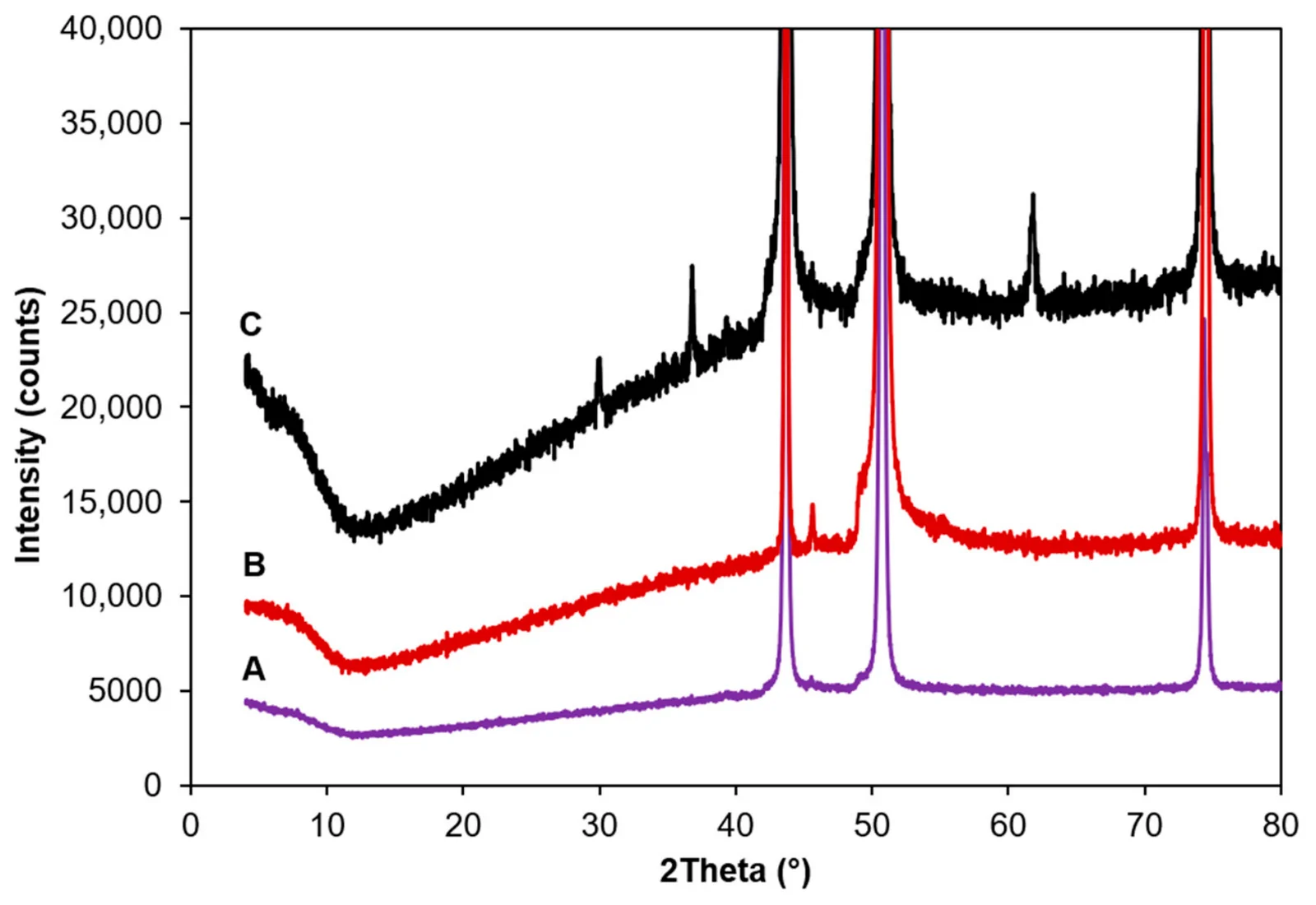
Zoomed XRD patterns of Cu samples: pure Cu (A), Cu with 20 CA layers after corrosion (B) and pure Cu after corrosion in 0.1 M NaCl (C).

**Figure 16:**
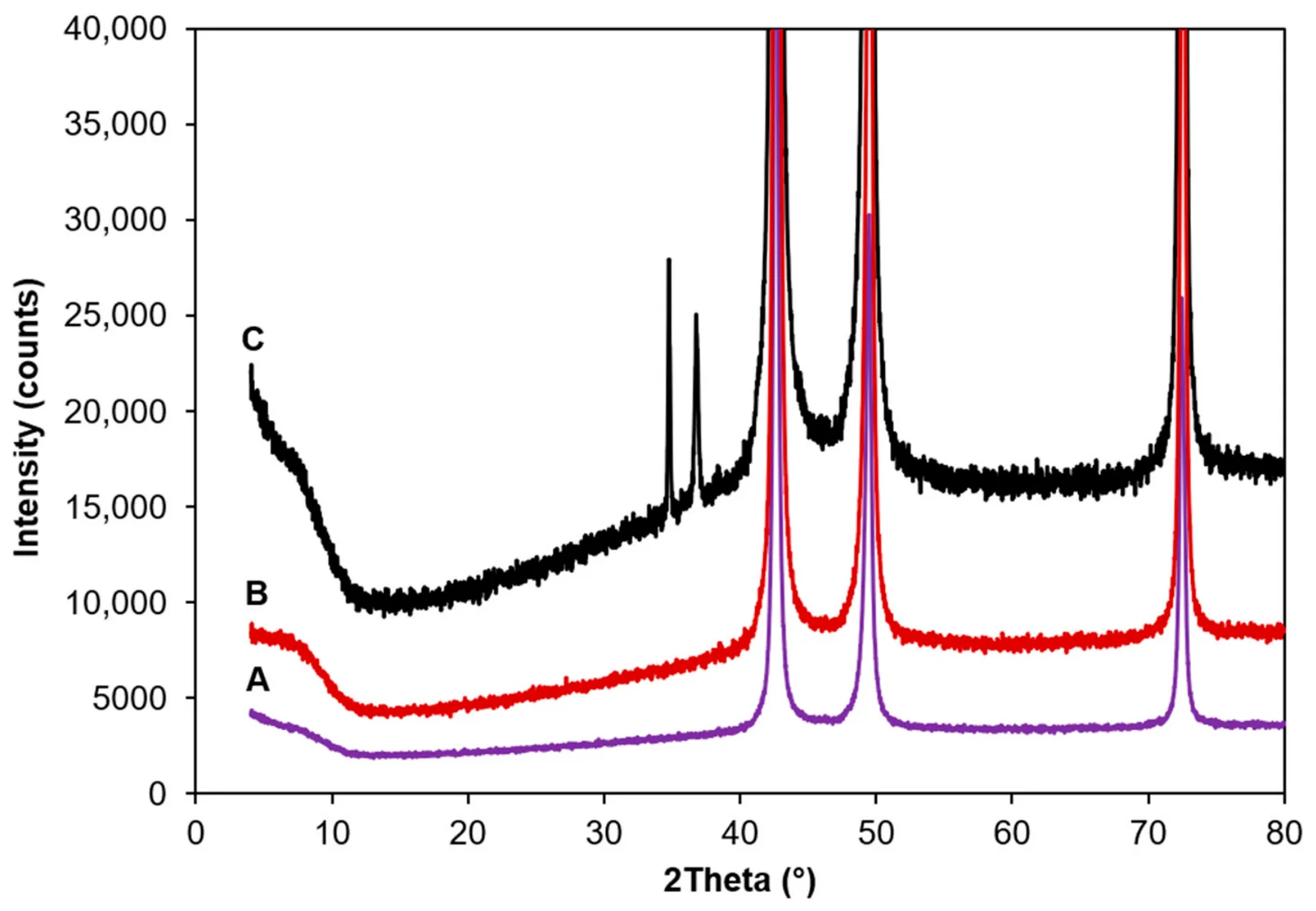
Zoomed XRD patterns of brass samples; pure brass (A), brass with 20 CA layers after corrosion (B) and pure brass after corrosion in 0.1 M NaCl (C).

The phase composition of the investigated samples was examined by powder X-ray diffraction (PXRD). The corresponding diffraction patterns are presented in Figure 15 and Figure 16 for copper and brass, respectively. The diffraction pattern of the untreated copper substrate (Figure 15 A) is characteristic of crystalline metallic Cu, with the main reflections observed at approximately 2θ = 43.3°, 50.4°, and 74.1°, corresponding to the (111), (200), and (220) crystallographic planes of the face-centered cubic (fcc) copper structure (JCPDS PDF-2 card no. 00-004-0836). After exposure to 0.1 M NaCl solution (Figure 15 C), an additional diffraction peak appears at approximately 2θ = 36.5°, which can be assigned to the (111) reflection of Cu_2_O (JCPDS PDF-2 card no. 01-071-3645). The formation of Cu_2_O confirms that partial oxidation of the copper surface occurred during the corrosion process. Cu_2_O is commonly reported as the primary corrosion product formed on copper under mildly corrosive chloride-containing environments. The diffraction patterns recorded for the brass samples (Figure 16) exhibit the characteristic reflections of the α-brass phase located at approximately 2θ = 42.3°, 49.3°, and 72.2° (JCPDS PDF-2 card no. 00-050-1333). Following immersion in NaCl solution, additional diffraction peaks appear at approximately 34° and 36.5°. These reflections are attributed to mixed copper–zinc oxide phases, indicating simultaneous oxidation of both alloy constituents. The considerably higher zinc content in the mixed oxide suggests that zinc undergoes preferential dissolution and oxidation, which is consistent with the well-known dezincification mechanism of brass corrosion in chloride media. During this process, zinc is selectively removed from the alloy surface, leaving behind a copper-enriched layer. In contrast, no additional diffraction peaks corresponding to crystalline corrosion products were detected for either the copper or brass specimens protected with the CA coating after immersion in 0.1 M NaCl solution. The XRD patterns of the coated samples remain essentially identical to those of the respective metallic substrates, indicating that the CA layer effectively suppresses the formation of detectable oxide phases. These results demonstrate that the deposited caffeic acid coating provides efficient protection against chloride-induced corrosion by acting as a barrier that limits the interaction between the aggressive electrolyte and the metal surface.

**Figure 17:**
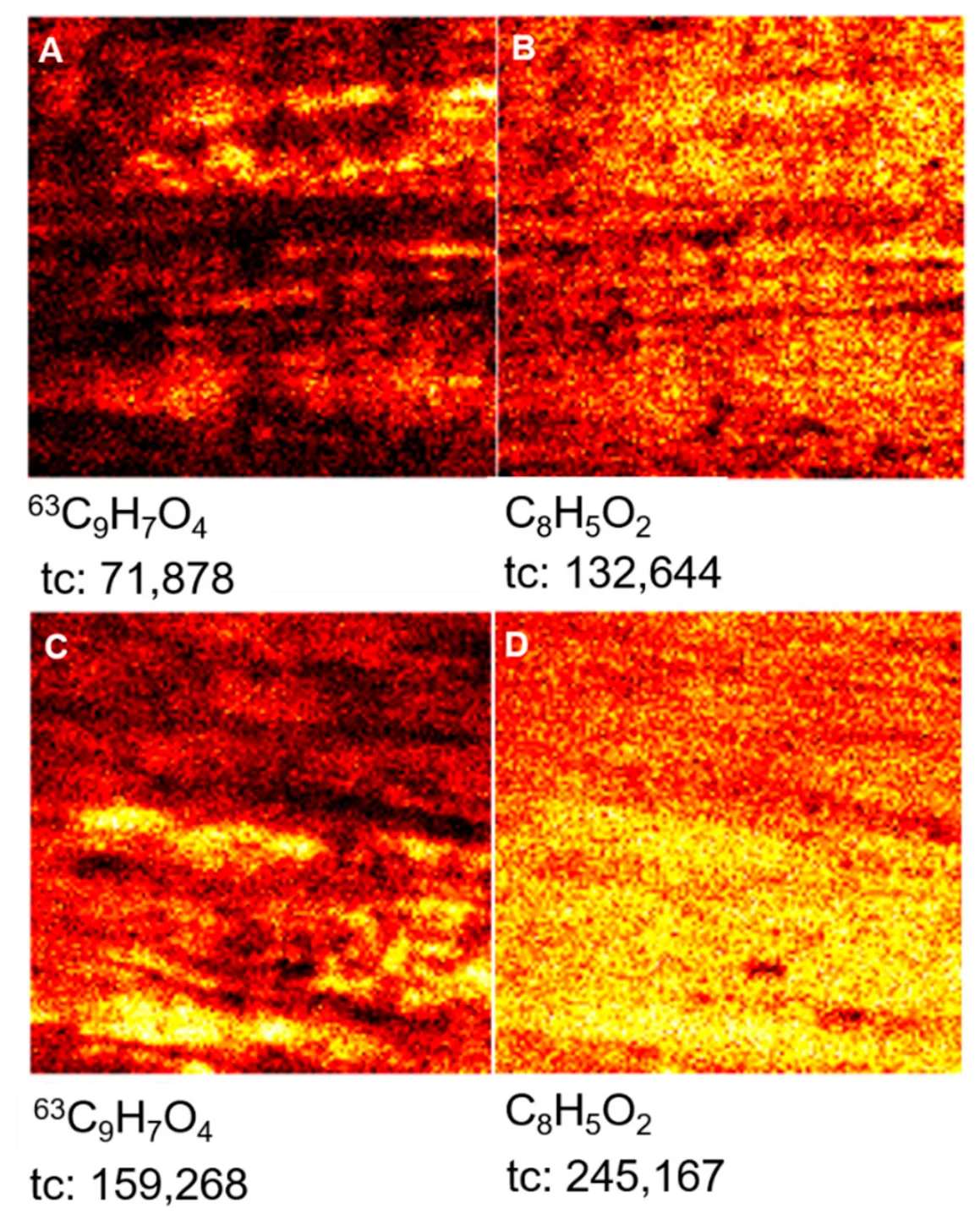
TOF-SIMS images of the Cu (**A**,**B**) and brass (**C**,**D**) samples covered with CA layers.

The corrosion products and presence of CA layers on samples surfaces were confirmed via TOF-SIMS analyses. TOF-SIMS images of Cu and brass surfaces covered with CA coating are presented in Figure 17.

Both TOF-SIMS images of Cu and brass (Figure 17) covered with CA coating exhibit non-uniform layers of CA on their surfaces, probably due to some scratches resulting from sample preparation before measurements. Two fragment ions, C_9_H_7_O_4_^−^ and C_8_H_5_O_2_^−^, visible on TOF-SIMS images, prove the presence of CA coating. The intensities of signals ascribed to these ions are approximately two times higher for brass in comparison to Cu. This is according to the fact that although both samples were covered with 20 layers of CA, the concentration of CA solution in the case of brass was two times higher (10 mM) than for Cu (5 mM). These concentrations were selected as optimal based on the electrochemical measurement results described in Section 2.1.2.

**Figure 18:**
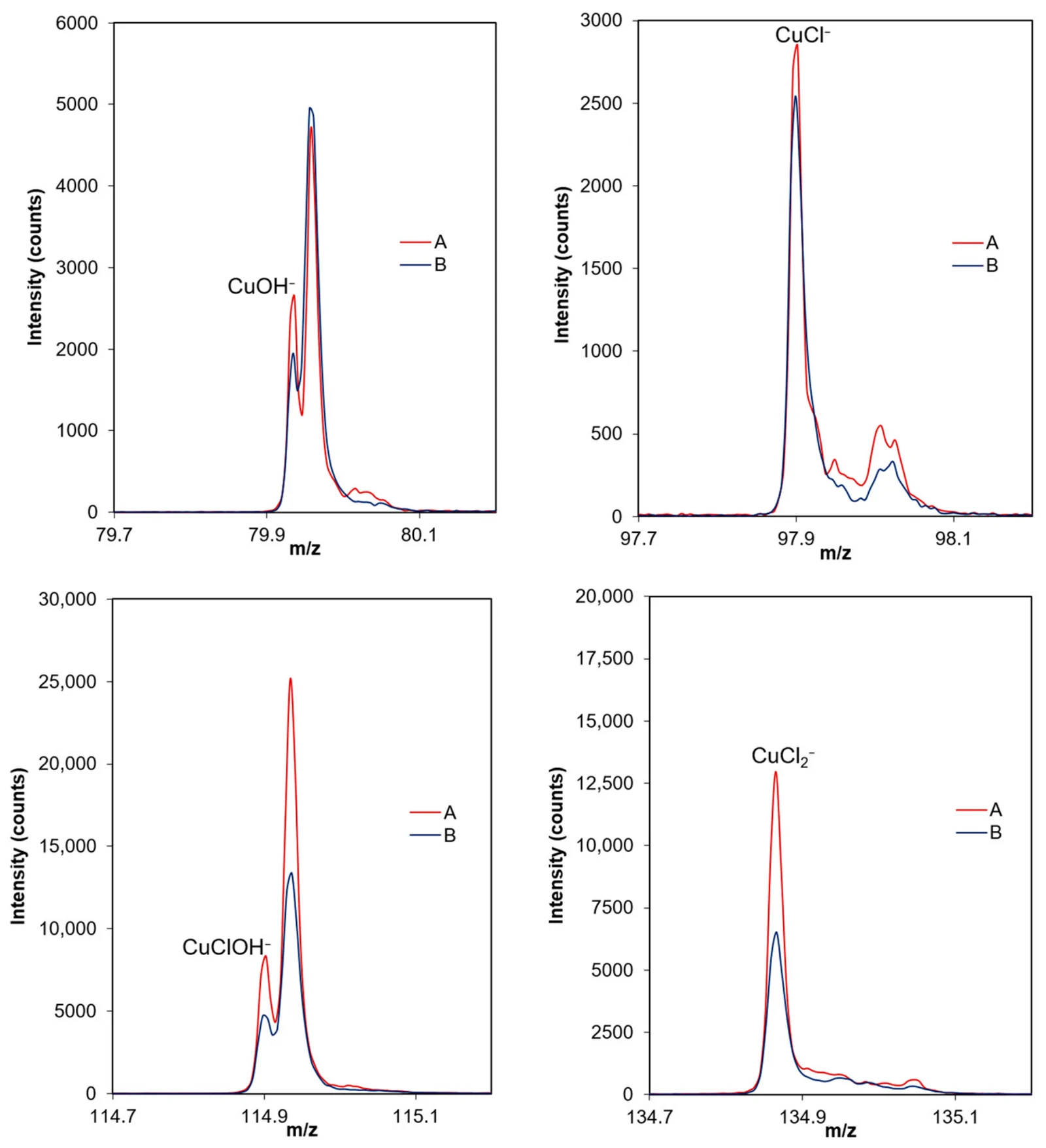
Exemplary TOF-SIMS spectra of the pure Cu sample (A) and samples coated with CA layers (B), both after immersion in NaCl.

The protective properties of CA for Cu were assessed based on the comparison of the spectra and images presented in Figure 18 and Figure S10. The emission of CuOH^−^, CuCl^−^, CuClOH^−^ and CuCl_2_^−^ ions at *m*/*z* 79.93, 97.90, 114.90 and 134.87, respectively, were chosen as indicative signals of Cu corrosion products formed according to Reactions (2)–(5) and additionally the formation of basic copper chloride Cu_2_(OH)_3_Cl. The possible formation of Cu_2_(OH)_3_Cl was confirmed and described in [66]. The intensities of signals are noticeably higher for Cu samples without CA layers, confirming the protective properties of CA coating. However, TOF-SIMS images reveal unevenly distributed corrosion products, indicating the possible occurrence of pitting corrosion.

**Figure 19:**
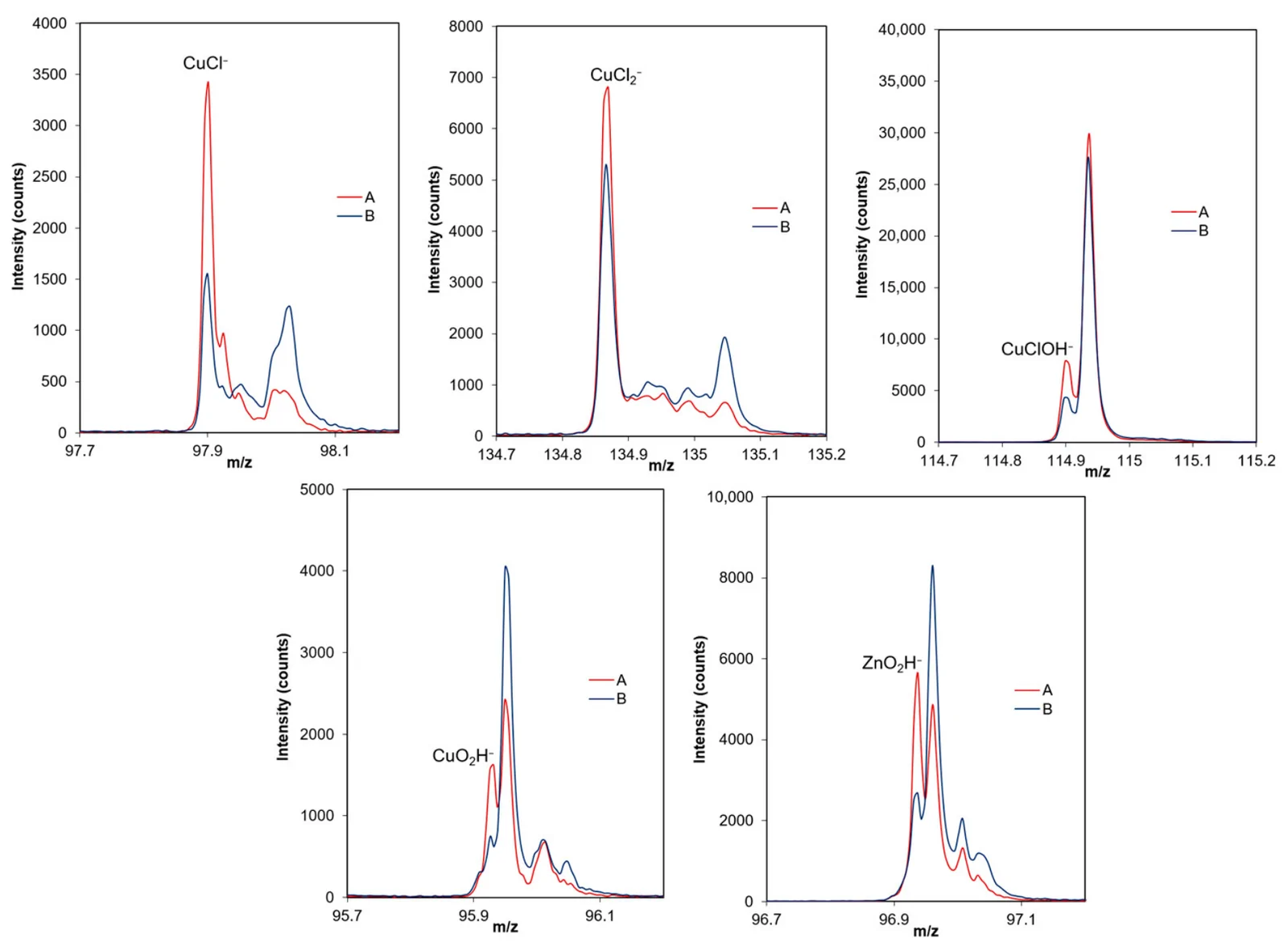
TOF-SIMS spectra of the pure brass sample (A) and samples coated with CA layers (B), both after immersion in NaCl.

On the other hand, CuCl^−^, CuCl_2_^−^, CuClOH^−^, CuO_2_H^−^, and ZnO_2_H^−^ ions (*m*/*z* 97.90, 134.87, 114.90, 95.93, and 96.94, respectively) were selected to represent the expected corrosion products for brass (Figure 19 and Figure S11).

The spectra presented in Figure 19 prove the presence of corrosion products formed in Reactions (7)–(11). Similarly to Cu, a formation of copper hydroxychloride Cu_2_(OH)_3_Cl was observed. The TOF-SIMS results for brass shown in Figure 19 and Figure S11 reveal considerably lower intensities of signals ascribed to brass corrosion products in the case of samples with CA coating.

The results presented above confirm the inhibitive properties of CA coatings for both Cu and brass samples immersed in NaCl medium. Additionally, it can be noticed that corrosion products forming on samples surfaces are not evenly distributed, which may indicate the occurrence of pitting corrosion.

## 3. Materials and Methods

### 3.1. Materials Preparation

**Figure 20:**
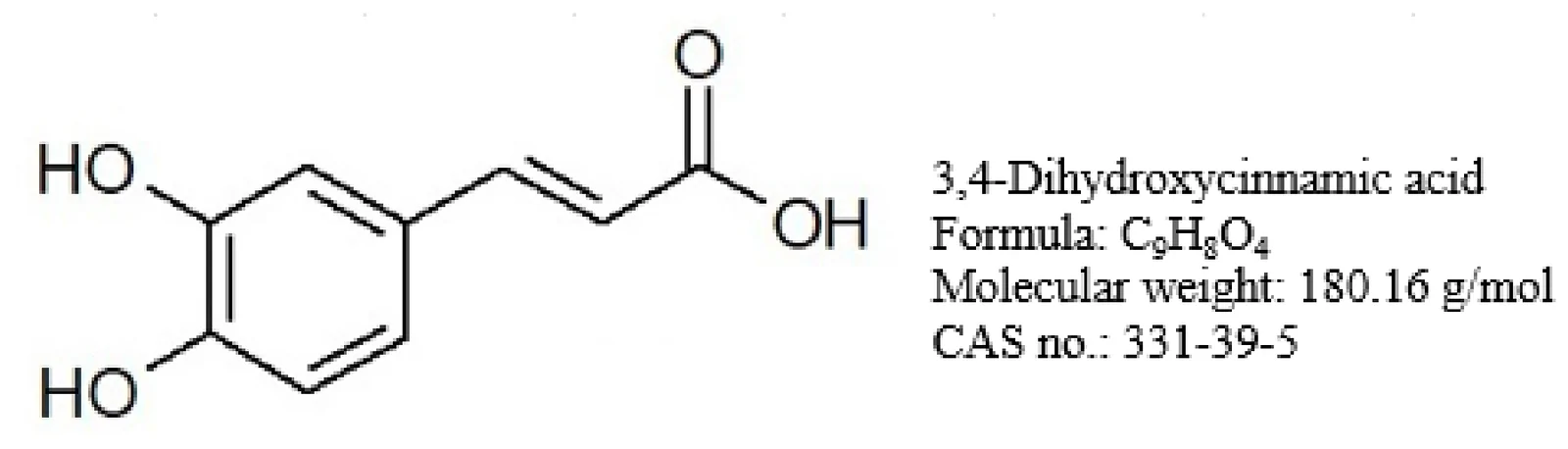
The structure of caffeic acid.

Electrolytic copper and brass M63 (63% Cu, 37% Zn) were used to conduct experiments. Each sample had an exposed surface of 1 cm^2^ and 1 mm thickness. Samples prior to each measurement were grinded with K180 sandpaper (LUX Tools, Wermelskirchen, Germany) and then degreased with acetone (99% Chempur, Piekary Slaskie, Poland). NaCl solution was prepared by dissolving NaCl (99.9% Chempur) powder in doubly distilled water. Caffeic acid solutions were made by dissolving the calculated mass of caffeic acid (Figure 20) powder (98%, Sigma-Aldrich, Saint Louis, MO, USA) in ethanol (99.9%, Supelco, Bellefonte, PA, USA).

Coatings of caffeic acid were formed on tested samples by brushing them with ethanol solutions of CA of different concentrations (5, 10, 20 mM). After the application of each layer, samples were dried in warm air.

### 3.2. Electrochemical Measurements

Electrochemical experiments were performed with the application of Autolab PGSTAT 302N potentiostat (Metrohm Autolab B.V., Utrecht, The Netherlands), which was controlled by NOVA 2.1.7 software.

All conducted electrochemical techniques were carried out in a four-electrode system. The silver chloride electrode (Ag/AgCl) was used as a reference electrode, two steel rods were used as auxiliary electrodes, while Cu and brass plates acted as working electrodes. All measurements were performed at room temperature.

Each PDP measurement was preceded by 1 h open circuit potential measurement. This was done to achieve a steady open circuit potential. Afterwards, samples were polarized in the range of OCP ± 0.3 V with a scan rate of 1 mV/s. The dependencies obtained from PDP measurements (j = F(E)) were then converted into Tafel plots (logj = F(E)) with the application of NOVA 2.1.7. software. From Tafel plots, basic corrosion parameters such as the corrosion current density (j_corr_), corrosion potential (E_corr_) and cathodic (b_c_) and anodic (b_k_) Tafel slopes were obtained. The corrosion rate (CR) was calculated according to the following equation [91]:

(20) $$\mathrm{CR}=\frac{j_{\mathrm{corr}}\cdot K\cdot \mathrm{EW}}{\rho }$$

where K—constat equals to 3270 μm (A cm year) ^−1^; EW—equivalent weight of metal (g mol^−1^); and ρ—metal density (g cm^−3^). The inhibition efficiency of CA (IE) was calculated using the following equation:

(21) $$\mathrm{IE}=\frac{j_{\mathrm{corr}}-j_{\mathrm{corr}(\mathrm{inh})}}{j_{\mathrm{corr}}}\cdot 100$$

where j_corr(inh)_ is the corrosion current density of the sample tested in the presence of CA.

EIS measurements were conducted at the OCP value by applying a sinusoidal signal with an applied amplitude of 10 mV, in the frequency range of 0.001–100,000 Hz. EIS results are shown as Nyquist plots and were used to determine not only the corrosion and inhibition mechanisms but also the kinetics of corrosion process. Data acquired from EIS measurements were analyzed using NOVA 2.1.7 software.

From EIS measurements, IE was calculated according to the following equation:

(22) $$\mathrm{IE}=\frac{R_{p(\mathrm{inh})}-R_{p}}{R_{p(\mathrm{inh})}}\cdot 100$$

### 3.3. Surface Characterization of Cu and Brass Samples

The changes in morphology of tested Cu and brass samples before and after immersion in NaCl as well as with and without CA coatings were investigated with the application of SEM-EDS, XRD and TOF-SIMS techniques.

Samples of copper and brass with and without CA coating were tested in the salt spray chamber model SF/MP/200 CW Specialist Equipment at 35 °C, with compressed air of 18–20 psi. Tested samples were kept at a 20° angle with continuous exposure to 5% NaCl spray as per ASTM B117. The SEM-EDX analyses of samples after salt spray exposure were performed with the scanning electron microscope FEI Quanta 650 (Thermo Fisher Scientific, Waltham, MA, USA).

The surface morphology of Cu and brass samples was investigated using scanning electron microscopy (SEM, S-4700, Hitach, Tokyo, Japan) coupled with energy-dispersive X-ray analysis (EDS, Noran System, Thermo Fisher Scientific, Waltham, MA, USA). Images are presented at ×500 magnification with a scale bar of 100 μm and ×1000 magnification with a scale bar of 50 μm. The applied voltage was 10 kV in the case of samples analyzed after salt spray chamber treatment and 25 kV for other measurements.

The secondary ion mass spectra were recorded using a TOF-SIMS IV mass spectrometer (IONTOF GmbH, Muenster, Germany). The instrument was equipped with a Bi liquid metal ion gun and high mass resolution time of flight mass analyzer. Secondary ion mass spectra were recorded for an approximately 100 × 100 μm^2^ area of the sample surface. During spectrum acquisition, the analyzed area of the sample surface was irradiated with pulses of 25 keV Bi^3+^ ions at a 10 kHz repetition rate. The average current of the primary ion pulsed beam was 0.2 pA, and the pulse duration was 1 ns. The time of spectrum acquisition was 30 s giving an ion dose below a static limit of 1 × 10^13^ ions cm^−2^. Ion sputtering with 3 keV Cs^+^ ions for 50 s was used to remove CA layers in order to perform the TOF-SIMS analysis of chemical composition of the sample below CA layers after corrosion in NaCl solution. Similarly, the spectra of the samples without CA layers were recorded from the sample surface cleaned by sputtering with 3 keV Cs^+^ ions for 50 s prior to the measurements. The current of the Cs^+^ beam was 13 nA and the sputtered area was 150 × 150 μm^2^. A pulsed 20 eV electron flood gun was used for charge neutralization during spectra acquisition.

## 4. Conclusions

The corrosion inhibition performance of caffeic acid (CA), a potential environmentally friendly corrosion inhibitor, was systematically investigated for copper (Cu) and brass in 0.1 M NaCl solution using mainly CA-based protective coatings, and for comparison purposes, by addition of CA to corrosive medium as its modification. The corrosion protection mechanisms and effectiveness of both approaches were evaluated using potentiodynamic polarization (PDP) and electrochemical impedance spectroscopy (EIS), supported by surface characterization and salt spray chamber testing. The results demonstrate that CA can effectively reduce the corrosion of both Cu and brass, although its performance depends on the substrate, inhibitor concentration, number of layers in a coating, and exposure duration.

When applied as a modification of the corrosive medium, CA provided maximum inhibition efficiencies of 72% for Cu at a concentration of 0.1 mM and 73% for brass at a concentration of 0.01 mM. These findings demonstrate that the optimal CA concentration is substrate-dependent, with the maximum inhibition efficiency for brass obtained for a concentration one order of magnitude lower than that required for Cu as well as for Zn, as was proved in our previous investigations. The relatively small changes in corrosion potential observed in the PDP measurements indicate that CA acts as a mixed-type corrosion inhibitor, affecting both anodic metal dissolution and cathodic reactions. The experimental data were fitted with the Langmuir adsorption isotherm, suggesting that the inhibition mechanism involves the adsorption of CA molecules onto the metal surfaces and is consistent with the formation of a monolayer. In the case of both tested samples, calculated ΔG_ads_ values indicate that the CA adsorption occurs via the mixed physisorption–chemisorption process. In particular, the value of ΔG_ads_ for Cu (−22 kJ mol^−1^) suggests that the participation of physisorption is significantly higher than chemisorption in contrast to Zn (−39.5 kJ mol^−1^). ΔG_ads_ for brass (−28.15 kJ mol^−1^) was between values calculated for Zn and Cu, indicating a clear mixed adsorption process. Increasing the CA concentration beyond the respective optimum resulted in a slight decrease in inhibition efficiency, indicating that higher inhibitor concentrations do not necessarily provide improved protection and may promote less favorable adsorption conditions or partial desorption. Prolonged immersion in the corrosive medium resulted in a slight improvement in inhibition efficiency for Cu, whereas a decrease was observed for brass, highlighting differences in the stability and evolution of the protective interfacial layers on the two substrates.

Clearly higher inhibition efficiencies were observed in the case of applying protective coatings formed from CA solutions, with the optimal coating parameters again depending on the substrate. For Cu, the highest inhibition efficiency of 87% was obtained using 20 layers of CA deposited from a 5 mM solution. For brass, the maximum inhibition efficiency of 81% was achieved using 20 layers formed from a 10 mM CA solution. CA-based coatings were more advantageous for Cu and brass under the investigated conditions. Furthermore, prolonged immersion in NaCl solution resulted in increased or stabilized inhibition efficiencies for the coated samples, suggesting that the protective properties of the coatings did not deteriorate during the investigated exposure time.

The EIS results corroborated the trends observed in the PDP measurements, confirming the effectiveness of both CA-based protection methods. In addition, the satisfactory description of the impedance spectra required equivalent electrical circuits incorporating a Warburg element, indicating that diffusion-related processes contributed to the electrochemical response under the investigated conditions. This finding suggests that mass transport, potentially involving the transport of electroactive species through the substrate/solution interface, plays a role in the corrosion process. However, the specific contribution of diffusion to the overall corrosion mechanism requires further investigation.

Surface characterization confirmed the presence of CA on both Cu and brass samples and supported the formation of corrosion products during exposure to the NaCl solution. Importantly, substantially fewer corrosion products were observed on the coated samples, providing additional evidence of the protective effect of the CA layers. The results of the salt spray chamber experiments further supported the corrosion protection afforded by the coatings, demonstrating that their effectiveness was not only proved by electrochemical experiments results.

The findings demonstrate that achieving effective corrosion protection with CA requires careful optimization of the protection method. For modification of the corrosive medium, the inhibitor concentration must be selected according to the substrate, whereas for protective coatings, both the CA concentration used during coating preparation and the number of deposited layers must be considered. Insufficient inhibitor concentrations or an inadequate number of layers forming a coating may provide limited protection. Therefore, the maximum concentration or number of layers does not necessarily correspond to the optimum corrosion protection.

Caffeic acid seems to be a promising green corrosion inhibitor for Cu and brass applied both as a corrosive medium modification and protective coatings. Additionally, the CA coatings did not visibly alter the color and the appearance of the protected samples, which is an important advantage for applications where preserving the original appearance of metallic objects is essential. These characteristics suggest potential applications in the conservation of historic and cultural heritage artefacts, particularly those made of copper or brass. Further investigations should address the long-term stability and durability of the coatings and their performance under various environmental conditions before they can be safely applied in the corrosion protection of historic artefacts.

## Figures and Tables

**Table 1:** The corrosion parameters obtained from Tafel plots recorded for Cu in 0.1 M NaCl modified by the addition of CA at various concentrations.

CA Conc. (mM)	E_corr_ (V)	b_a_ (V dec^−1^)	b_c_ (V dec^−1^)	j_corr_ (A cm^−2^)	CR (mm year^−1^)	IE (%)	Θ (-)
0	−0.273	0.137	−0.033	1.66 × 10^−6^	0.0193	-	-
0.001	−0.273	0.045	−0.029	1.49 × 10^−6^	0.0172	10.9	0.11
0.005	−0.279	0.046	−0.025	1.30 × 10^−6^	0.0151	27.8	0.28
0.01	−0.265	0.034	−0.019	7.22 × 10^−7^	0.0084	56.5	0.57
0.05	−0.266	0.048	−0.020	6.36 × 10^−7^	0.0074	61.7	0.62
0.1	−0.248	0.032	−0.018	4.78 × 10^−7^	0.0055	71.5	0.72
0.5	−0.171	0.036	−0.041	8.59 × 10^−7^	0.0100	48.2	0.48

**Table 2:** The corrosion parameters obtained from Tafel plots recorded for brass in 0.1 M NaCl modified by the addition of CA at various concentrations.

CA Conc. (mM)	E_corr_ (V)	b_a_ (V dec^−1^)	b_c_ (V dec^−1^)	j_corr_ (A cm^−2^)	CR (mm year^−1^)	IE (%)	Θ (-)
0	−0.220	0.043	−0.040	8.19 × 10^−7^	0.0102	-	-
0.0001	−0.205	0.039	−0.035	6.35 × 10^−7^	0.0079	22.5	0.23
0.0005	−0.227	0.031	−0.026	5.87 × 10^−7^	0.0073	30.3	0.30
0.001	−0.209	0.024	−0.024	3.62 × 10^−7^	0.0045	55.9	0.56
0.005	−0.204	0.020	−0.018	2.83 × 10^−7^	0.0035	65.7	0.66
0.01	−0.200	0.014	−0.014	2.26 × 10^−7^	0.0028	72.5	0.73
0.02	−0.204	0.022	−0.021	2.91 × 10^−7^	0.0036	64.7	0.65

**Table 3:** Adsorption parameters for CA on Cu and brass surfaces in 0.1 M NaCl.

Sample	K_ads_ (L mM^−1^)	ΔG_ads_ (kJ mol^−1^)	R^2^ (-)
Cu	114	−21.68	0.995
brass	1547	−28.15	0.997

**Table 4:** Corrosion parameters obtained from three consecutive measurements for Cu in 0.1 M NaCl with the addition of 0.1 mM of CA.

Measur. No.	E_corr_ (V)	b_a_ (V dec^−1^)	b_c_ (V dec^−1^)	j_corr_ (A cm^−2^)	CR (mm year^−1^)	IE (%)
**0.1 M NaCl**						
**1**	−0.273	0.137	−0.033	1.66 × 10^−6^	0.0193	-
**2**	−0.280	0.500	−0.102	4.64 × 10^−6^	0.0540	-
**3**	−0.281	0.647	−0.130	5.45 × 10^−6^	0.0632	-
**0.1 mM CA**						
**1**	−0.248	0.032	−0.018	4.78 × 10^−7^	0.0055	71.5
**2**	−0.255	0.050	−0.029	1.10 × 10^−6^	0.0128	76.3
**3**	−0.256	0.047	−0.028	1.12 × 10^−6^	0.0130	79.4

**Table 5:** Corrosion parameters determined from three consecutive measurements for brass in 0.1 M NaCl with the addition of 0.01 mM CA.

Measur. No.	E_corr_ (V)	b_a_ (V dec^−1^)	b_c_ (V dec^−1^)	j_corr_ (A cm^−2^)	CR (mm year^−1^)	IE (%)
**0.1 M NaCl**						
**1**	−0.220	0.043	−0.040	8.19 × 10^−7^	0.0102	-
**2**	−0.241	0.043	−0.031	8.72 × 10^−7^	0.0109	-
**3**	−0.261	0.035	−0.034	9.40 × 10^−7^	0.0117	-
**0.01 mM CA**						
**1**	−0.200	0.014	−0.014	2.26 × 10^−7^	0.0028	72.5
**2**	−0.207	0.025	−0.026	3.44 × 10^−7^	0.0043	60.6
**3**	−0.212	0.032	−0.031	4.05 × 10^−7^	0.0050	57.3

**Table 6:** EIS parameters of Cu samples in 0.1 M NaCl with and without the addition of CA.

CA Conc. (mM)	Χ^2^ (-)	R_s_ (Ω)	R_f+ox_ (Ω)	Q_f+ox_ (S·s) ^n^ _f+ox_	n_f+ox_	R_ct_ (Ω)	C_dl_ (μF)	W × 10^−4^ (Ω^−1^ cm^−2^ s^0.5^)	IE (%)
0	0.013	65.2	295.4	1.711 × 10^−3^	0.459	536.0	13.09	5.513	-
0.001	0.023	65.8	427.0	1.493 × 10^−3^	0.451	754.5	6.45	5.311	29.6
0.005	0.012	68.4	480.6	2.048 × 10^−3^	0.433	800.3	6.62	6.849	35.1
0.01	0.026	63.6	532.4	1.402 × 10^−3^	0.445	950.2	5.61	7.863	43.9
0.05	0.017	68.7	612.9	2.500 × 10^−3^	0.428	1024.5	4.51	10.83	49.2
0.1	0.052	72.1	846.0	3.191 × 10^−3^	0.442	1165.4	4.15	19.56	58.7
0.5	0.027	70.5	734.4	4.786 × 10^−3^	0.565	634.2	7.51	3.812	39.3

**Table 7:** EIS parameters of brass samples in 0.1 M NaCl with and without the addition of CA.

CA Conc. (mM)	Χ^2^ (-)	R_s_ (Ω)	R_f+ox_ (Ω)	Q_f+ox_ (S·s) ^n^ _f+ox_	n_f+ox_	R_ct_ (Ω)	C_dl_ (μF)	W × 10^−4^ (Ω^−1^ cm^−2^ s^0.5^)	IE (%)
0	0.006	69.6	2699.5	3.183 × 10^−4^	0.675	119.6	30.24	7.489	-
0.0001	0.047	67.3	5780.1	8.710 × 10^−5^	0.723	369.3	23.59	4.779	54.2
0.0005	0.026	69.3	6178.3	7.407 × 10^−5^	0.776	524.8	20.52	3.000	57.9
0.001	0.012	69.5	6836.4	7.997 × 10^−5^	0.765	430.0	23.48	3.316	61.2
0.005	0.018	70.6	6907.8	6.201 × 10^−5^	0.773	361.4	18.56	3.502	61.2
0.01	0.007	64.4	18,502.0	5.806 × 10^−5^	0.622	23.0	3.11	3.260	84.8
0.02	0.003	60.0	11,132.0	5.552 × 10^−5^	0.642	23.0	5.62	3.868	74.7

**Table 8:** Corrosion parameters obtained from Tafel plots recorded for Cu coated with various numbers of layers formed form CA solutions of different concentrations.

Number of Layers	E_corr_ (V)	b_a_ (V dec^−1^)	b_c_ (V dec^−1^)	j_corr_ (A cm^−2^)	CR (mm year^−1^)	R_p_ (Ω)	P (%)	IE (%)
5 mM CA								
1	−0.216	0.033	−0.029	4.83 × 10^−7^	0.0056	13,740	19.3	71.0
5	−0.207	0.033	−0.024	3.65 × 10^−7^	0.0042	16,296	14.0	78.2
10	−0.211	0.027	−0.021	3.20 × 10^−7^	0.0037	15,925	15.3	80.8
20	−0.205	0.020	−0.016	2.26 × 10^−7^	0.0026	16,779	12.8	86.5
30	−0.208	0.029	−0.021	3.16 × 10^−7^	0.0037	16,601	13.3	80.8
10 mM CA								
1	−0.227	0.033	−0.047	4.01 × 10^−7^	0.0046	20,930	15.2	76.2
5	−0.206	0.035	−0.024	3.49 × 10^−7^	0.0040	17,613	12.7	79.3
10	−0.207	0.031	−0.023	3.32 × 10^−7^	0.0039	16,987	13.4	79.8
20	−0.207	0.030	−0.023	3.71 × 10^−7^	0.0043	15,113	15.1	77.7
30	−0.210	0.036	−0.023	3.01 × 10^−7^	0.0035	20,186	11.7	81.9
20 mM CA								
1	−0.205	0.034	−0.024	3.71 × 10^−7^	0.0043	16,387	13.4	77.7
5	−0.204	0.041	−0.027	4.00 × 10^−7^	0.0046	17,825	12.1	76.2
10	−0.190	0.039	−0.028	5.51 × 10^−7^	0.0064	12,809	13.3	66.8
20	−0.214	0.030	−0.028	3.62 × 10^−7^	0.0042	17,635	14.7	78.2
30	−0.205	0.036	−0.024	3.28 × 10^−7^	0.0038	19,283	11.4	80.3

**Table 9:** Corrosion parameters obtained from Tafel plots recorded for brass in 0.1 M NaCl with various numbers of layers formed from CA solutions of different concentrations.

Number of Layers	E_corr_ (V)	b_a_ (V dec^−1^)	b_c_ (V dec^−1^)	j_corr_ (A cm^−2^)	CR (mm year^−1^)	R_p_ (Ω)	P (%)	IE (%)
5 mM CA								
1	−0.207	0.018	−0.018	3.34 × 10^−7^	0.0042	11,581	47.3	58.8
5	−0.209	0.017	−0.019	3.07 × 10^−7^	0.0038	12,795	47.7	62.7
10	−0.207	0.018	−0.017	3.35 × 10^−7^	0.0042	11,327	48.4	58.8
20	−0.208	0.018	−0.018	3.23 × 10^−7^	0.0040	12,037	38.8	60.8
30	−0.204	0.014	−0.014	2.28 × 10^−7^	0.0028	13,381	34.9	72.5
10 mM CA								
1	−0.204	0.018	−0.017	2.81 × 10^−7^	0.0035	13,578	34.4	65.7
5	−0.201	0.016	−0.015	2.70 × 10^−7^	0.0034	12,617	31.5	66.7
10	−0.205	0.013	−0.014	2.41 × 10^−7^	0.0030	11,418	43.1	70.6
20	−0.206	0.014	−0.015	1.56 × 10^−7^	0.0019	19,881	26.1	81.4
30	−0.207	0.018	−0.018	3.07 × 10^−7^	0.0038	12,946	42.4	62.7
20 mM CA								
1	−0.211	0.025	−0.025	4.84 × 10^−7^	0.0060	11,277	60.2	41.2
5	−0.221	0.031	−0.027	5.21 × 10^−7^	0.0065	12,093	86.2	36.3
10	−0.216	0.025	−0.026	4.22 × 10^−7^	0.0053	13,210	67.2	48.0
20	−0.213	0.016	−0.016	2.89 × 10^−7^	0.0036	12,075	62.6	64.7
30	−0.212	0.013	−0.013	3.04 × 10^−7^	0.0038	9523	75.2	62.7

**Table 10:** Corrosion parameters obtained from Tafel curves for Cu coated with 20 layers of CA (5 mM) conducted in 0.1 M NaCl.

Measur. No.	E_corr_ (V)	b_a_ (V dec^−1^)	b_c_ (V dec^−1^)	j_corr_ (A cm^−2^)	CR (mm year^−1^)	R_p_ (Ω)	P (%)	IE (%)
1	−0.205	0.020	−0.016	2.26 × 10^−7^	0.0026	16,779	12.8	86.5
2	−0.207	0.032	−0.024	3.91 × 10^−7^	0.0045	15,472	5.5	91.7
3	−0.284	0.068	−0.011	5.04 × 10^−7^	0.0058	8025	14.4	90.8

**Table 11:** Corrosion parameters obtained from Tafel curves for brass with 20 layers of CA (10 mM) conducted in 0.1 M NaCl.

Measur. No.	E_corr_ (V)	b_a_ (V dec^−1^)	b_c_ (V dec^−1^)	j_corr_ (A cm^−2^)	CR (mm year^−1^)	R_p_ (Ω)	P (%)	IE (%)
1	−0.206	0.014	−0.015	1.56 × 10^−7^	0.0019	19,881	26.1	81.4
2	−0.202	0.012	−0.012	1.63 × 10^−7^	0.0020	16,514	6.7	81.7
3	−0.204	0.014	−0.015	2.05 × 10^−7^	0.0026	15,268	1.2	77.8

**Table 12:** EIS parameters obtained for Cu samples in 0.1 M NaCl with different numbers of layers formed from 5 mM CA solution.

Number of Layers	Χ^2^ (-)	R_s_ (Ω)	R_coat_ (Ω)	Q_coat_ (S·s) ^n coat^	n_coat_	R_ct_ (Ω)	C_dl_ (μF)	W × 10^−4^ (Ω^−1^ cm^−2^ s^0.5^)	IE (%)
0	0.013	65.2	295.4	1.711 × 10^−3^	0.459	536.0	13.09	5.513	-
1	0.006	71.0	1802.9	7.148 × 10^−4^	0.613	33.3	18.76	16.20	54.7
5	0.004	70.0	2628.9	4.888 × 10^−4^	0.733	86.4	13.25	4.911	69.4
10	0.007	69.6	3101.8	4.544 × 10^−4^	0.687	104.1	13.44	5.340	74.1
20	0.011	69.6	3524.7	4.802 × 10^−4^	0.693	85.4	13.81	5.813	77.0
30	0.020	72.6	2893.2	6.362 × 10^−4^	0.694	56.1	15.22	7.494	71.8

**Table 13:** EIS parameters of brass samples in 0.1 M NaCl with different numbers of layers formed form 10 mM CA solution.

Number of Layers	Χ^2^ (-)	R_s_ (Ω)	R_coat_ (Ω)	Q_coat_ (S·s)^n coat^	n_coat_	R_ct_ (Ω)	C_dl_ (μF)	W × 10^−4^ (Ω^−1^ cm^−2^ s^0.5^)	IE (%)
0	0.006	69.6	2699.5	3.183 × 10^−4^	0.675	119.6	30.24	7.489	-
1	0.037	68.8	5351.5	4.929 × 10^−5^	0.808	1811.6	53.14	3.050	60.6
5	0.002	71.0	7404.0	1.070 × 10^−4^	0.784	105.2	19.48	2.441	62.5
10	0.031	70.4	6753.0	6.155 × 10^−5^	0.783	1438.0	52.73	1.993	65.6
20	0.007	74.6	9660.3	7.029 × 10^−5^	0.611	168.5	10.86	4.612	71.3
30	0.008	71.8	9668.2	8.470 × 10^−5^	0.597	78.5	6.42	4.739	71.1

**Table 14:** Elemental composition of Cu and Cu with 20 protective layers of CA, before and after immersion in 0.1 M NaCl.

Element	wt. (%)	
	Cu	Cu after Immersion
C	9.59	9.57
O	0.51	4.73
Al	0.86	2.87
Cu	89.04	82.83
	Cu 20 layers	Cu 20 layers after immersion
C	15.24	14.31
O	1.11	0.85
Al	0.57	0.78
Cu	83.08	84.05

**Table 15:** Elemental composition of brass and brass with 20 protective layers of CA, before and after immersion in 0.1 M NaCl.

Element	wt. (%)	
	Brass	Brass after Immersion
C	11.18	14.38
O	0.00	6.61
Al	1.62	3.70
Cu	56.94	48.03
Zn	30.25	27.29
	brass 20 layers	brass 20 layers after immersion
C	17.45	18.92
O	1.69	2.65
Al	0.85	0.90
Cu	52.29	51.88
Zn	27.72	25.65

## Data Availability

The data is contained within the article.

## Supplementary Materials

The following supporting information can be downloaded at https://www.mdpi.com/article/10.3390/molecules31213837/s1. Figure S1: Tafel plots recorded for three consecutive measurements for Cu in 0.1 M NaCl with the addition of 0.1 mM CA. Figure S2: Tafel plots recorded for three consecutive measurements for brass in 0.1 M NaCl with the addition of 0.01 mM CA. Figure S3: Tafel plots recorded for three consecutive measurements for Cu coated with 20 layers of CA (5 mM) conducted in 0.1 M NaCl. Figure S4: Tafel plots recorded for three consecutive measurements for brass coated with 20 layers of CA (10 mM) conducted in 0.1 M NaCl. Figure S5: EDS mapping of Cu (A and B) and brass (C and D) surfaces with (right side) and without CA coating (left side) corresponding to SEM images presented in Figure 15 A,C (Cu), and Figure 16 A,C (brass). Figure S6: EDS spectra for Cu sample (A), Cu after immersion in NaCl (B), Cu with 20 layers of caffeic acid before (C) and after immersion in NaCl (D). Figure S7: EDS spectra for brass sample (A), brass after immersion in NaCl (B), brass with 20 layers of caffeic acid before (C) and after immersion in NaCl (D). Figure S8: EDS mapping of pure Cu (A) and Cu with 20 layers of CA (B) after a week-long exposure to NaCl in salt spray chamber. Figure S9: EDS mapping of brass (A) and brass with 20 layers of CA (B) after a week-long exposure to NaCl in salt spray chamber. Figure S10: Exemplary TOF-SIMS spectra and images of the pure Cu sample (A) and samples coated with CA layers (B) both after immersion in NaCl. Figure S11: TOF-SIMS images of pure brass samples (A) and samples covered with CA layers (B) both after immersion in NaCl.
