# Supplementary material for: Electrochemical Evaluation of Caffeic Acid as an Eco-Friendly Corrosion Inhibitor for Brass and Copper in NaCl
Source: Molecules. 2026 Oct 23;31(21):3837. doi: 10.3390/molecules31213837 (PMC13764780; doi:10.3390/molecules31213837)
Supplement: Supplementary file 1 [file molecules-31-03837-s001.zip › molecules-4640174-supplementary.pdf]

Supplementary Information

Electrochemical Evaluation of Caffeic Acid as an Eco-Friendly Corrosion Inhibitor for Brass and Copper in NaCl

Aleksander Kucharek [1,*], Elżbieta Kuśmierek [1,*], Aleksandra Bednarek [1], Aleksandra Zimon [1], Waldemar Maniukiewicz [1], Kashif Mairaj Deen [2] and Małgorzata Iwona Szynkowska-Jóźwik [1]

[1] Institute of General and Ecological Chemistry, Lodz University of Technology, ul. Zeromskiego 116, 90-924 Lodz, Poland; aleksandra.zimon@p.lodz.pl (A.Z.); waldemar.maniukiewicz@p.lodz.pl (W.M.);

[2] Department of Materials Engineering, The University of British Columbia, Vancouver, BC V6T 1Z4, Canada; kashifmairaj.deen@ubc.ca

* Correspondence: aleksander.kucharek@dokt.p.lodz.pl (A.K.); elzbieta.kusmierek@p.lodz.pl (E.K.)

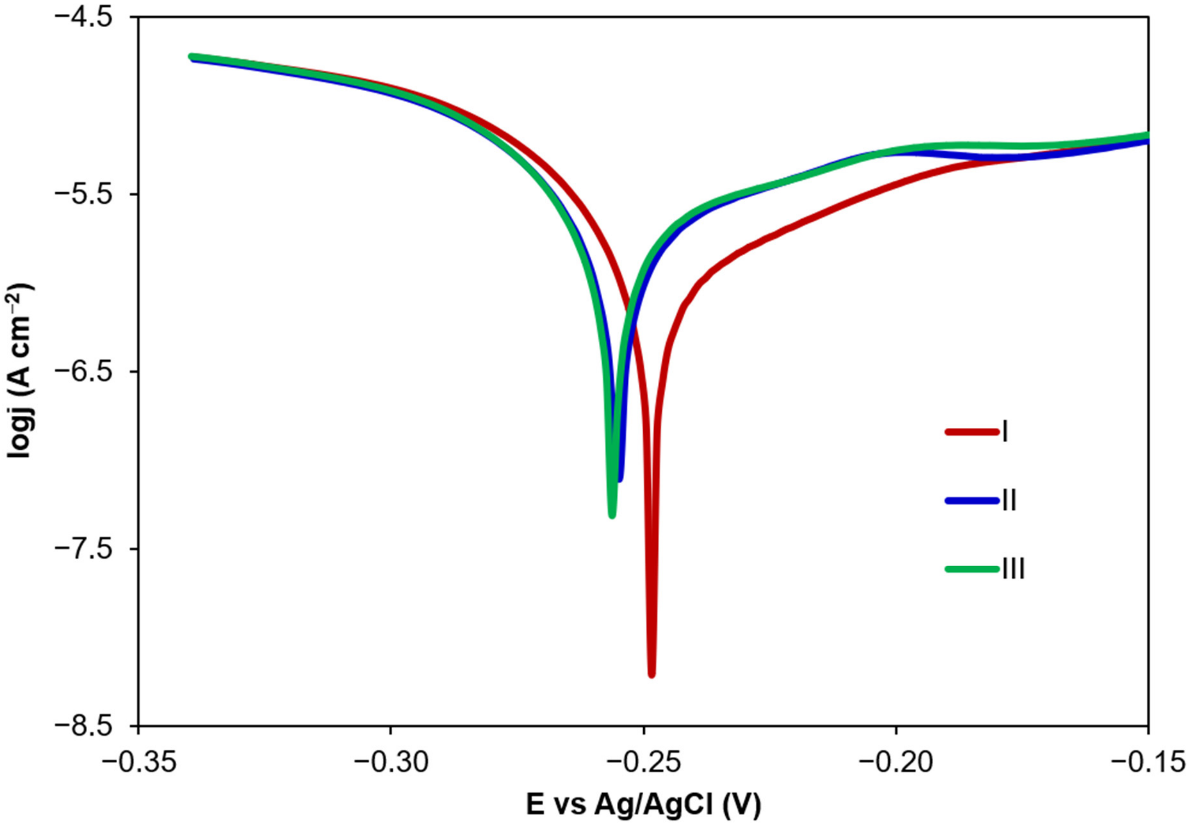

Figure S1. Tafel plots recorded for three consecutive measurements for Cu in 0.1 M NaCl with the addition of 0.1 mM CA.

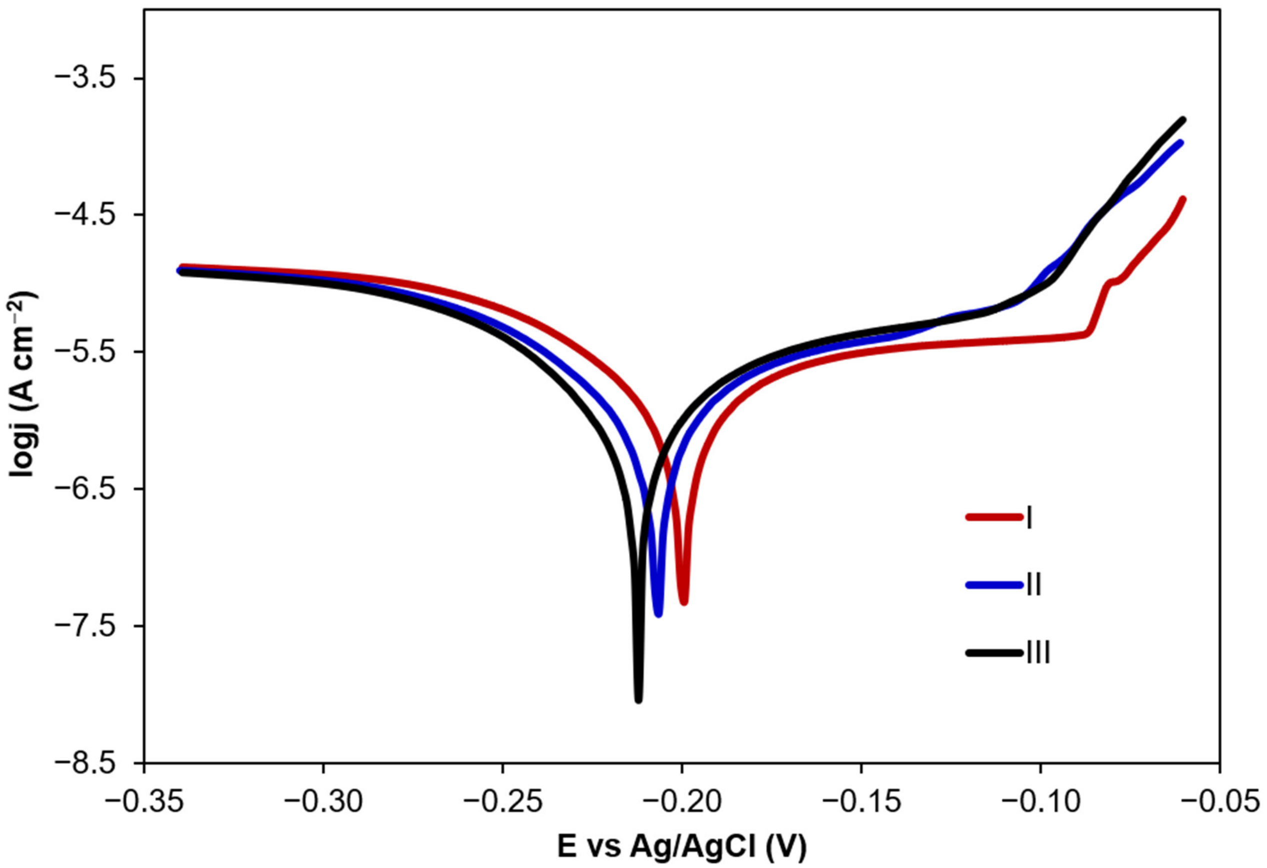

Figure S2. Tafel plots recorded for three consecutive measurements for brass in 0.1 M NaCl with the addition of 0.01 mM CA.

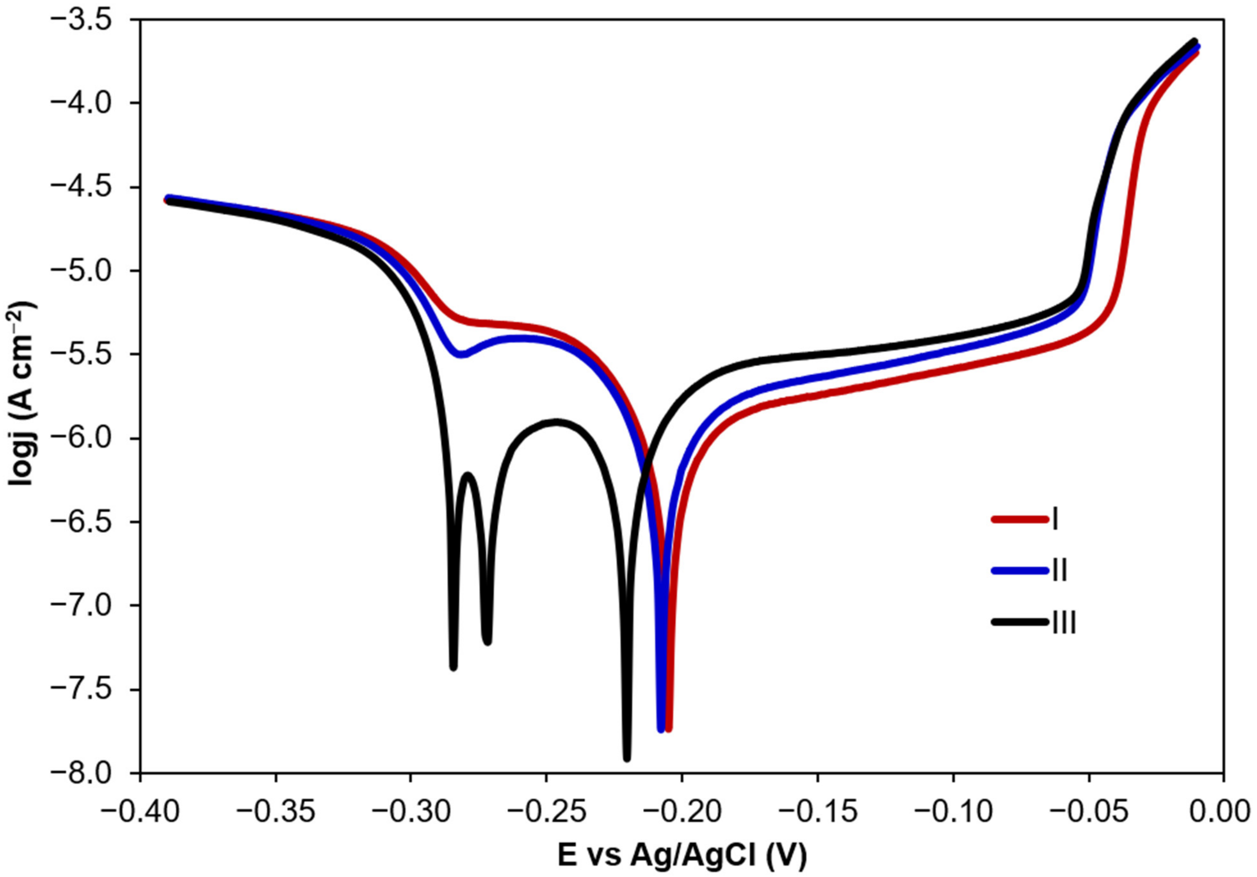

Figure S3. Tafel plots recorded for three consecutive measurements for Cu coated with 20 layers of CA (5 mM) conducted in 0.1 M NaCl.

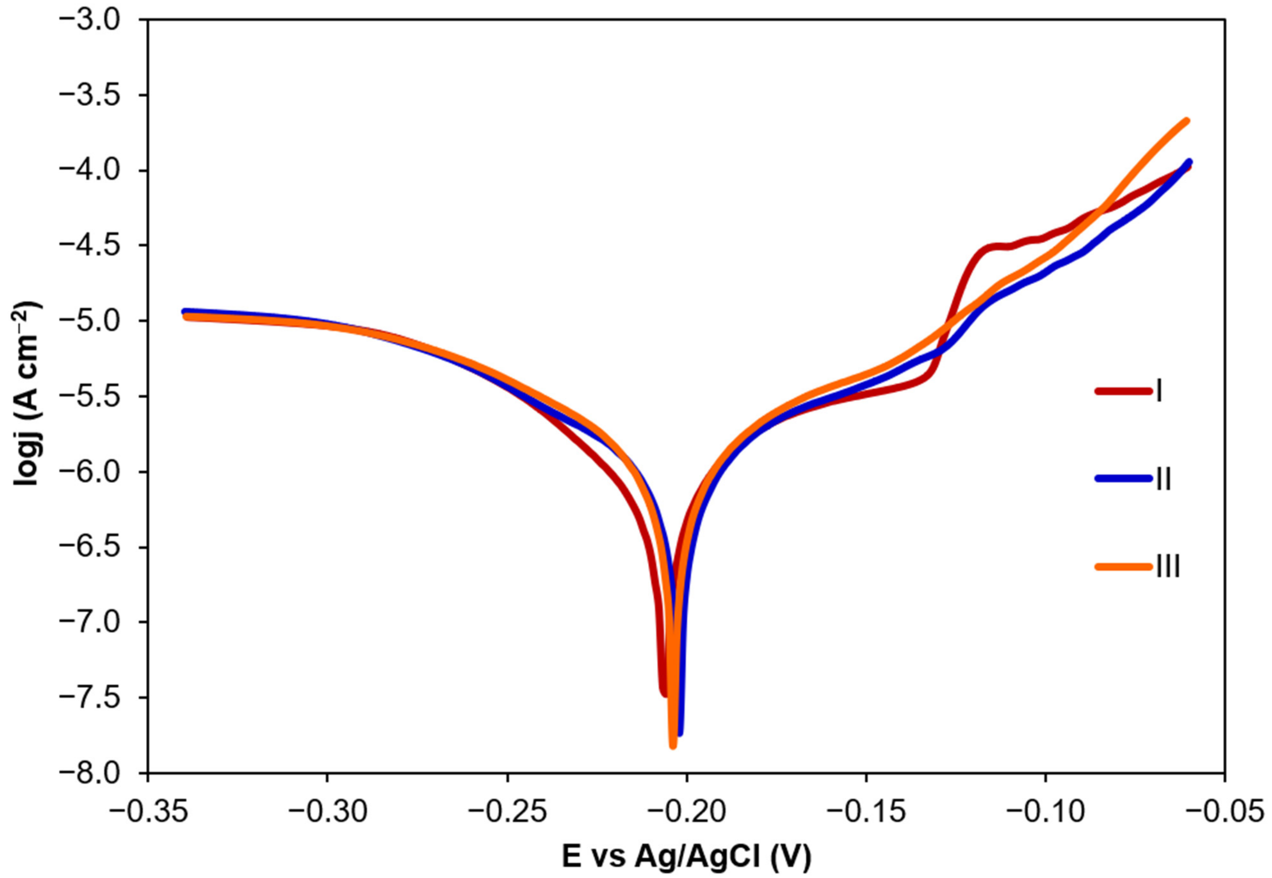

Figure S4. Tafel plots recorded for three consecutive measurements for brass coated with 20 layers of CA (10 mM) conducted in 0.1 M NaCl.

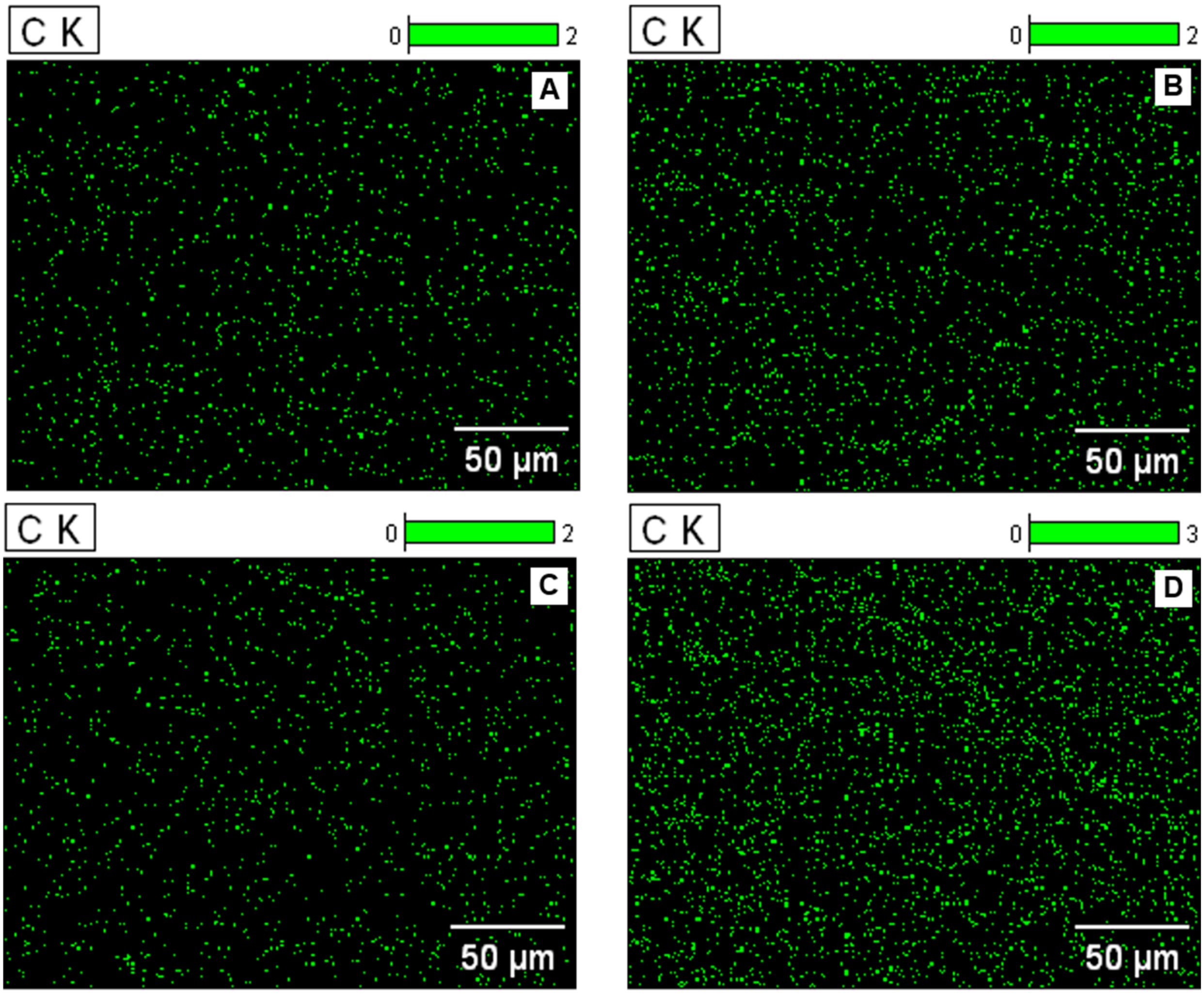

Figure S5. EDS mapping of Cu (A and B) and brass (C and D) surfaces with (right side) and without CA coating (left side) corresponding to SEM images presented in Figure 15A and 15C (Cu), and Figure 16A and 16C (brass).

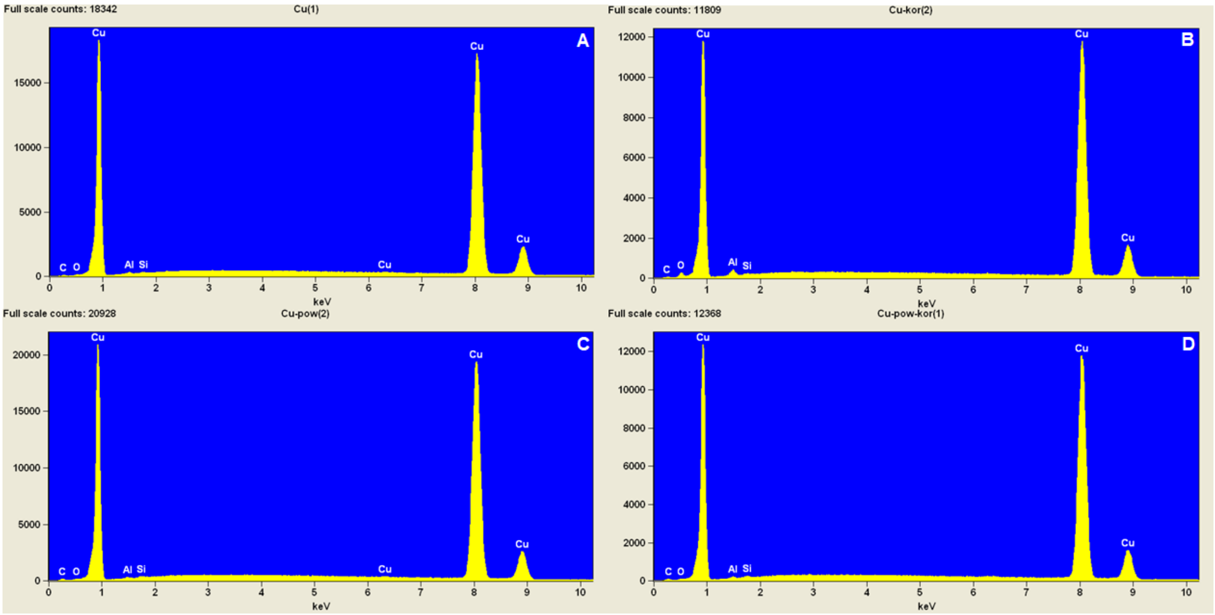

Figure S6. EDS spectra for Cu sample (**A**), Cu after immersion in NaCl (**B**), Cu with 20 layers of caffeic acid before (**C**) and after immersion in NaCl (**D**).

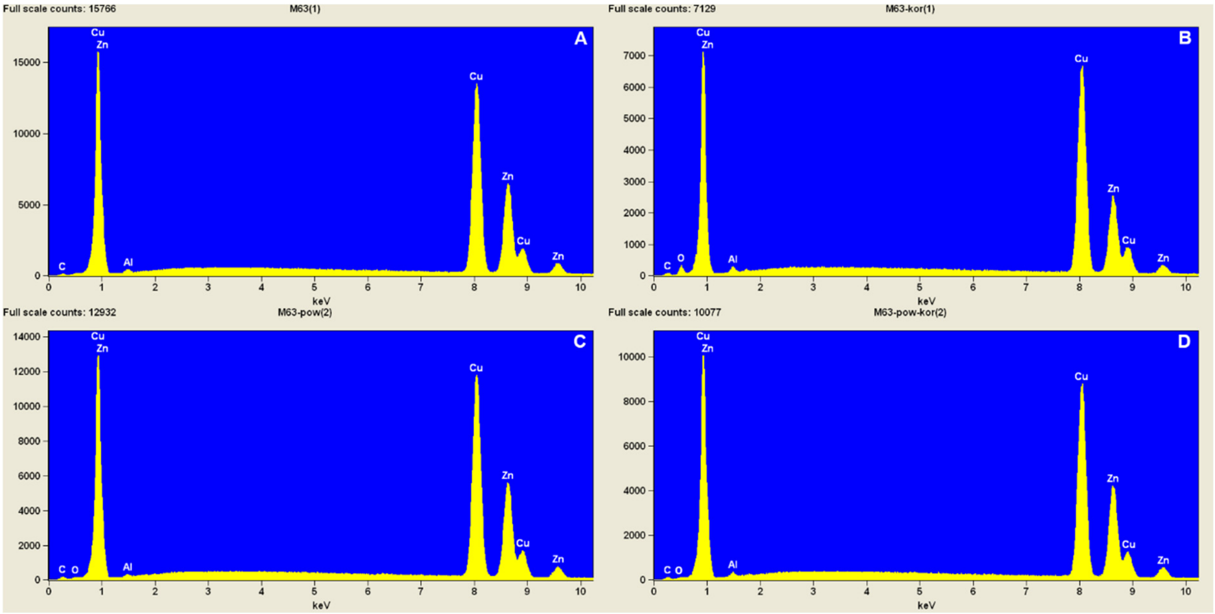

Figure S7. EDS spectra for brass sample (**A**), brass after immersion in NaCl (**B**), brass with 20 layers of caffeic acid before (**C**) and after immersion in NaCl (**D**).

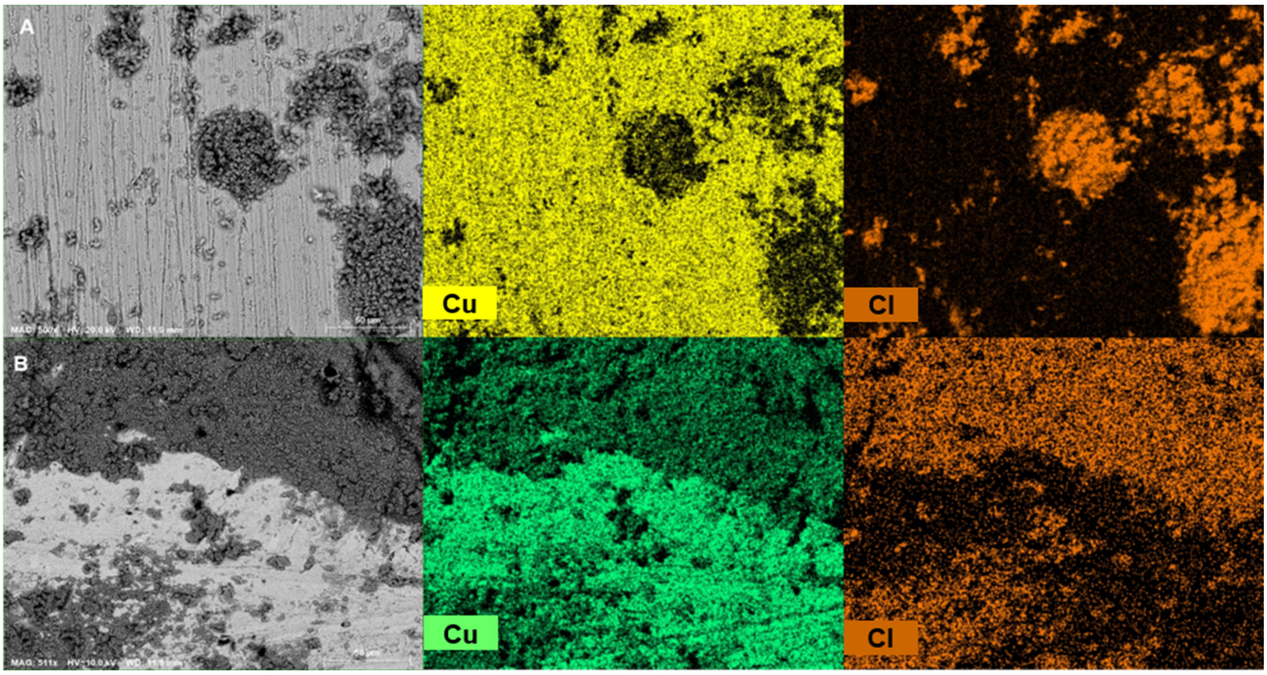

Figure S8. EDS mapping of pure Cu (**A**) and Cu with 20 layers of CA (**B**) after a week-long exposure to NaCl in salt spray chamber.

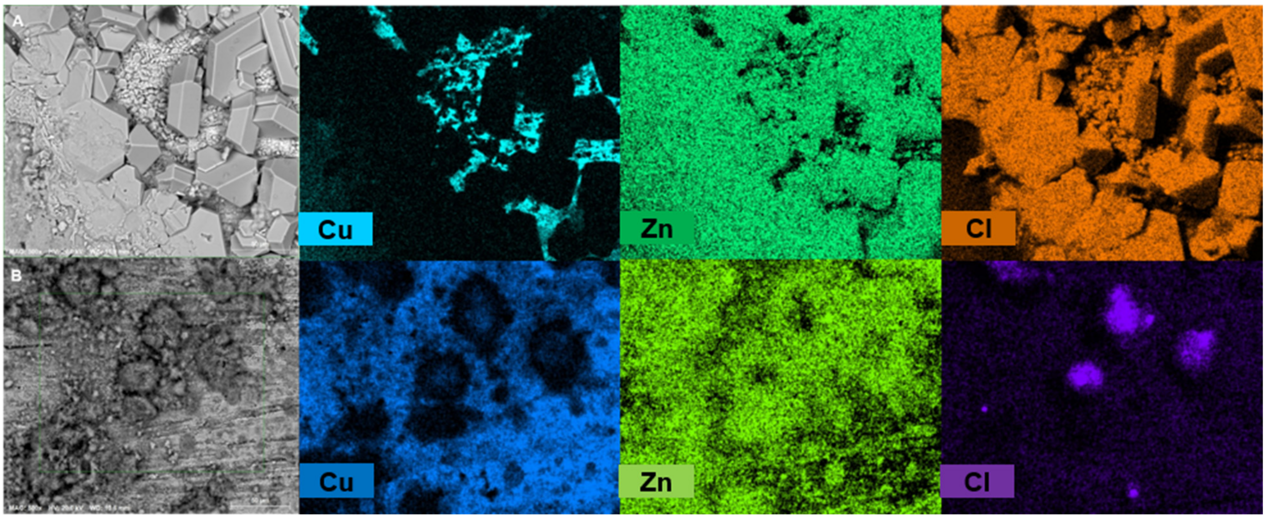

Figure S9. EDS mapping of brass (**A**) and brass with 20 layers of CA (**B**) after a week-long exposure to NaCl in salt spray chamber.

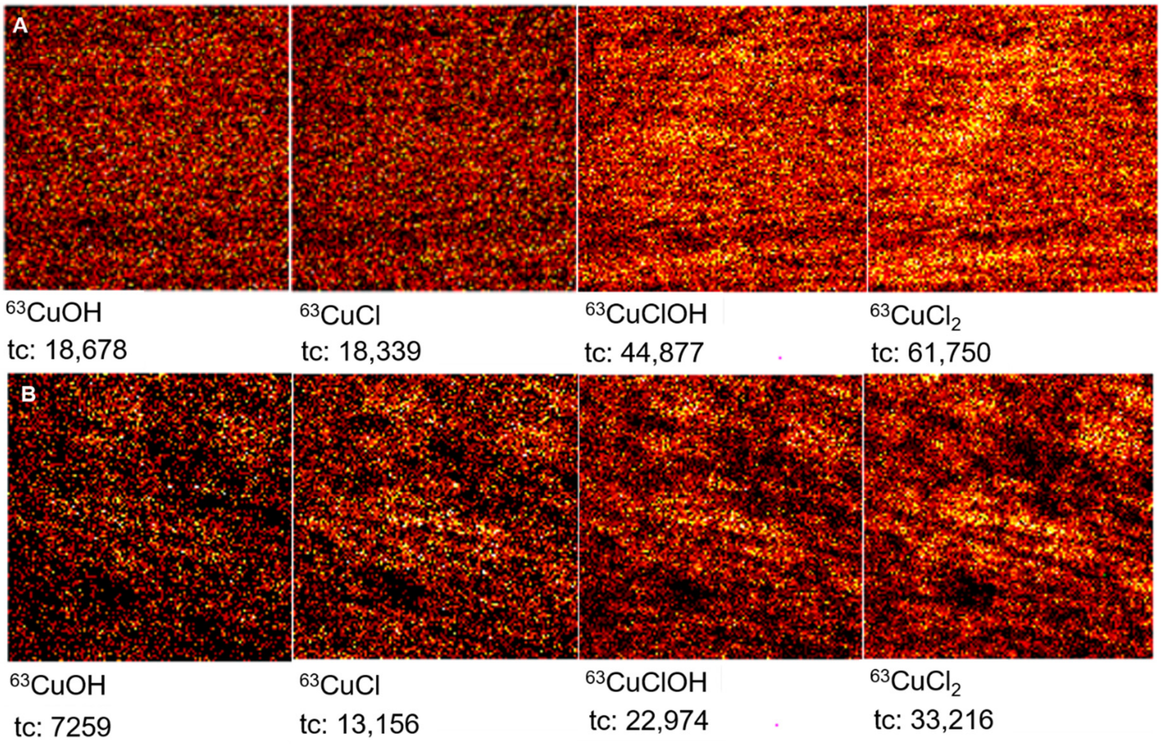

Figure S10. Exemplary TOF-SIMS spectra and images of the pure Cu sample (A) and samples coated with CA layers (B) both after immersion in NaCl.

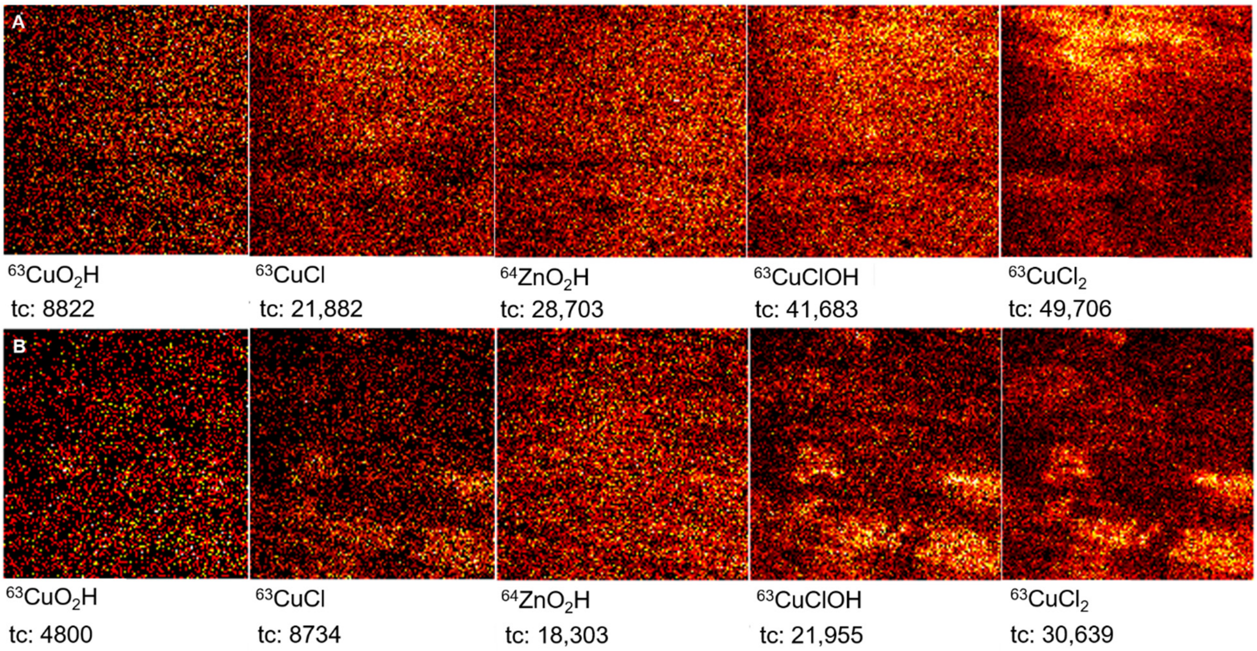

Figure S11. TOF-SIMS images of pure brass samples (A) and samples covered with CA layers (B) both after immersion in NaCl.
